# Conference Proceedings – 4^th^ International Conference on Molecular Diagnostics and Biomarker Discovery: Antibody Technology: Penang, Malaysia 25^th^ – 26^th^ September 2019

**DOI:** 10.1186/s12919-019-0169-6

**Published:** 2019-12-10

Published on

## I-01 Welcome to the 4^th^ International Conference on Molecular Diagnostics and Biomarker Discovery: Antibody Technology

### Rahmah Noordin (rahmah@usm.my) (Advisor: 4^th^ International Conference on Molecular Diagnostics and Biomarker Discovery: Antibody Technology)

#### Institute for Research in Molecular Medicine, Universiti Sains Malaysia, 11800 USM, Penang, Malaysia

**Organization:** Institute for Research in Molecular Medicine, Universiti Sains Malaysia.

**Support:** Higher Institution Centre of Excellence (HICoE), Ministry of Education Malaysia

Universiti Sains Malaysia

**Funding Support**

The conference and this publication was funded by Ministry of Education Malaysia, Higher Institution Centre of Excellence (HICoE), under the grant (HICoE: 311/CIPPM/4401005)

**Organizing Committee**

Yee Siew Choong (Chair)

Gee Jun Tye

Noral Wiah Hj Abdul Karim

Theam Soon Lim

Khairul Mohd Fadzli Mustaffa

Chiuan Herng Leow

Chiuan Yee Leow

Venugopal Balakrishnan

Rahmah Noordin (Advisor)

**Venue**

Equatorial Hotel Penang, Penang, Malaysia

The Institute for Research in Molecular Medicine (INFORMM) is a research institute under the Universiti Sains Malaysia. It was established in the year 2003 as a translational and multidisciplinary institute, with strengths in research on diagnostics and biomarker discovery. It aims to provide diagnostics solutions, especially to those affecting people in low resource settings. There are three research clusters at INFORMM, i.e., Diagnostics for Infectious Diseases (DID), Advanced Research Technologies (ART), and Cancer Research (CARE). In the year 2010, INFORMM gained recognition by the Malaysian Ministry of Education as one of the country’s Higher Institution Centre of Excellence (HICoE), in the niche area of Diagnostics Platform.

Molecular Diagnostics and Biomarker Discovery (MDBD) is an international conference held annually by INFORMM since the year 2016, with support from the Ministry of Education. The conference provides a platform for scientists and postgraduate students locally and internationally to share their new findings, and deliberate on the current topics, as well as to network and initiate collaborations. The three research clusters at INFORMM take turns to organize the annual MDBD conference. This year, the ART cluster led the organization of the 4th MDBD with the theme of “Antibody Technology.” Universiti Sains Malaysia is also celebrating its 50th anniversary; thus, the organization of this conference with minimal registration fees showed the university’s commitment to advancing science and technology.

Antibody technology is a platform that transcends various areas of research, whether Diagnostics, Vaccines, or Therapeutics. With the recent breakthroughs in immunotherapy, biologics are set to lead the way in the treatment of diseases. As one of the most dominant biologic format, monoclonal antibodies stand ready to capitalize on this. Building on two decades of research, it offers exciting advancements in the treatment of communicable and non-communicable diseases ranging from cancer to autoimmunity to infectious diseases. The development of antibody technology also benefitted the development of diagnostics, especially in reducing the time required for an antibody to go from bench to bedside and increasing the test specificity. The conference also focused on alternative binders that mimic antibodies such as DNA/RNA aptamers and other non-antibody scaffolds. The size of these non-antibody scaffolds and its specificity rivals that of an antibody and could potentially be used hand in hand with antibodies for both diagnostics and therapeutics.

The 2019 MDBD attracted 95 participants, including international participants from Thailand, Indonesia, Kazakhstan, Arab Saudi, and India. There were ten invited speakers from eight countries, i.e., Germany, Singapore, Thailand, Arab Saudi, Denmark, South Africa, Korea, and the USA. The abstracts of the conference published in the BMC Proceedings reflect the diversity of the research papers presented.

## IS01 Invited Speaker - Targeting Tyrosine Kinases, Tubulin and Topoisomerase for Cancer Therapy

### Malose J. Mphahlele (mphahmj@unisa.ac.za)

#### Department of Chemistry, University of South Africa, Private Bag X06, Florida 1710, South Africa

**Background**

Cancer is responsible for increase in the mortality rate and has become a life threatening disease affecting people at all ages in both developing and developed countries. There are several types of cancer treatment and these include surgery, radiation therapy, chemotherapy and targeted therapy each with its advantages and disadvantages. Targeted therapy is the foundation of precision medicine and it makes use of small molecules that can attach to specific targets inside or on the outer surface of cancer cells. Our focus towards compounds with potential anticancer properties has previously been limited to their evaluation for cytotoxicity in vitro against panel of cancer cell lines. However, cytotoxicity does not define a specific cellular death mechanism. There are several mechanisms of action for the anticancer agents including induction of apoptosis, DNA and mitochondrial damage, inhibition of angiogenesis, tubulin inhibition, kinase inhibition, and also drug efflux protein activities- or a combination of some of these mechanisms. We have since extended our research on heterocyclic compounds with potential anticancer properties to include their mechanism of anticancer activity.

**Methodology**

The prepared compounds are screened for antigrowth effect against panel of cancer cell lines using the 3-(4,5-dimethylthiazol)-2,5-diphenyl tetrazolium bromide (MTT) assay. Selected compounds are then evaluated for potential to induced apoptosis by means of flow cytometry and caspase activation assays. Non-cell based assays are conducted on the most active compound for inhibitory effects s against tubulin polymerization or protein kinases and topoisomerase I/II enzymes.

**Results and Discussion**

In our previous investigations on the cytotoxicity of polysubstituted indoles and the 4-anilinoquinazolines, it was observed that these compounds induce apoptosis. Their mechanisms of anticancer activity as potential inhibitors of epidermal growth factor receptor tyrosine kinases (EGFR-TK) [1] or tubulin polymerization [2] were evaluated experimentally complemented with molecular docking (*in silico*) into the ATP binding site of EGFR or colchicine tubulin binding site, respectively. In a quest to optimize this class of potential anti-proliferative agents, the aniline group was replaced with a heterocyclic framework such as indole or benzofuran ring to comprise quinazoline-appended hybrids and the compounds were evaluated for anticancer activity in vitro against a panel of cancer cell lines and for their potential to inhibit EGFR-TK phosphorylation complemented. A series of benzo[*c*]furan-chalcones was synthesized and evaluated *in vitro* for antigrowth effect and for dual inhibitory effect against tubulin polymerization and EGFR-TK phosphorylation [3].

**Conclusion**

The N- and O-containing heterocycles prepared in our laboratory and their molecular hybrids have been found to be cytotoxic and to induce apoptosis in various cancer cell lines. Some of these compounds were found to inhibit tubulin polymerization or EGFR-TK phosphorylation. The experimental results were complemented with molecular docking into the active sites of these receptors to help rationalize the anticancer activity and guide further structure activity relationship (SAR) studies.

**References**

[1] Mphahlele, M.J. et al. *Pharmaceuticals* 2017, *10*, 87.

[2] Mphahlele, M.J.; Parbhoo, N. *Pharmaceuticals* 2018, *11*, 59.

[3] Mphahlele, M.J. et al. *Int. J. Mol. Sci.* 2018, *19*, 2552.

## IS02 Invited Speaker - Recombinant antivenoms based on broadly-neutralizing sweeping human monoclonal antibodies

### Andreas Hougaard Laustsen (ahola@bio.dtu.dk)

#### Department of Biotechnology and Biomedicine, Technical University of Denmark, Søltofts Plads 224, DK-2800 Kongens Lyngby, Denmark

**Background**

Snakebite envenoming is a neglected tropical disease affecting 2.5 million victims worldwide each year, leading to more than 100,000 deaths and about 400,000 amputations and other sequalae. Currently, snakebite envenoming is treated with polyclonal antivenoms comprising heterologous antibodies derived from the plasma of immunized horses. Due to their non-human nature, such antivenoms have a propensity to cause adverse reactions in human recipients, as well as their content of therapeutically active antibodies is low. Therefore, snakebite victims, when treated, receive exorbitantly high amounts of immunogenic animal antibodies, which both represents an issue for safety and cost of treatment [1].

**Methodology**

Recombinant antivenoms based on mixtures of human monoclonal antibodies can be designed to have improved therapeutic properties, such as enhanced efficacy, better safety profiles, and improved manufacturability compared to existing animal plasma-derived polyclonal antivenoms. These next generation antivenoms may therefore have the potential to replace existing plasma-derived antivenoms [2].

**Results and Discussion**

In the later years, significant scientific developments have been reported within the field of recombinant antivenoms, including the development of strategies for broadening the neutralization capacity of toxin-neutralizing antibodies and the generation of the first experimental recombinant antivenom based on an oligoclonal mixture of fully human monoclonal immunoglobulin G antibodies [3]. However, many technical challenges still lie ahead before recombinant antivenoms may enter clinical development. These challenges include how to generate broadly-neutralizing antibodies that can neutralize multitudes of toxins, and how to design efficacious antibody mixtures that can be administered at very low dose. Here, novel strategies for the discovery and engineering of broadly-neutralizing sweeping antibodies are presented with examples of such antibodies against snake, spider, scorpion, and bee toxins recently discovered in my lab.

**Conclusion**

Recent progress in the field of recombinant antivenoms has created renewed hope that novel antibody discovery methodologies and recombinant DNA technology may enable the development of polyvalent recombinant snakebite antivenoms that can be manufactured at low cost.

**References**

[1] Laustsen AH. How can monoclonal antibodies be harnessed against neglected tropical diseases and other infectious diseases? Expert Opin Drug Discov. 2019; ePub:1-10.

[2] Laustsen AH, et al. Recombinant snakebite antivenoms: A cost-competitive solution to a neglected tropical disease? PLoS Negl Trop Dis. 2017; 11:1-14.

[3] Laustsen AH, et al. *In vivo* neutralization of dendrotoxin-mediated neurotoxicity of black mamba venom by oligoclonal human IgG antibodies. Nat Commun. 2018; 9:1-9.

## Sessions

### OP02 Effects of Cinnamomum Zeylanicum (Cinnamon) on Oral Squamous Cell Carcinoma *In vivo*: An Immunohistochemical study

#### Aied M Alabsi^1^, Oon C. Shan^1^, Noraliaa Jaafar^1^, Mahani Mahdzir^1^, Lim X. Yun^1^, Darren Y. W. Xuan^1^, Rola Ali-Saeed^1^, May Alkoshab^2^

##### ^1^Faculty of Dentistry, MAHSA University, Bandar Saujana Putra, 42610 Jenjarom Kuala Langat, Selangor. Malayasia; ^2^Department of Oral and Craniofacial Sciences, Faculty of Dentistry, University of Malaya, 50603 Kuala Lumpur, Malaysia

###### **Correspondence:** Aied M Alabsi (aied@mahsa.edu.my)

**Background**

Oral cancer is a subtype of cancers that are commonly referred to as head and neck cancers. The cancer comprises about 85% of all the head and neck cancers. More than 90% of oral cancers are squamous cell carcinoma (OSCC) originating in the tissues, which line the lips, oral cavity and pharynx [1,2]. OSCC is characterized by a high degree of local invasiveness and high rate of metastasis to the cervical lymph nodes. Traditional herbal medicine played a significant role in cancer therapy throughout history. *Cinnamomum zeylanicum*, also known as Ceylon cinnamon or “true cinnamon”, is obtained from the bark of a tropical evergreen tree, an indigenous plant in Sri Lanka and grows widely in Madagascar, India, and Indo-China. The active compound of Cinnamon has been observed to have anti-microbial, anti-parasitic and anti-oxidant properties, reducing blood glucose level, cholesterol level, and blood pressure, anti-inflammatory activity and anticancer properties [3].

**Methodology**

In this study, *Cinnamomum Zeylanicum* was investigated for its cytotoxic effect on 4NQO-induced oral cancer carcinogenesis in rats tongue. Twenty SD rats were randomly divided into four equal groups; Group I received water as a negative control and Group II received 4NQO for 8 weeks at 20 ppm (positive control). Group III and IX were given 4NQO and treated with Cinnamon at 250 and 500 mg/kg, respectively. All rats from all experiments were sacrificed after 22 weeks, and the histopathological changes were evaluated. Furthermore, Immunohistochemical expressions of tumor markers; cyclin D1, β-catenin and e-cadherin were analysed in tongue specimens using an image analyser computer system.

**Results and Discussion**

The results showed that the Cinnamon extract reduced the incidence of OSCC when compared to the control group, especially in the high dose group. Histopathological results showed changes in 4NQO-induced cancer group without treatment, three of 5 tongue specimens were diagnosed as having OSCC while two of them diagnosed as having dysplastic lesions. Following treatment with the Cinnamon extract, three of 5 tongue specimens were diagnosed as having hyperplasia, one diagnosed as having dysplastic lesions and one specimen only have reached to OSCC stage. Immunohistochemistry staining results showed significant (p<0.05) up-regulation of β-catenin and e-cadherin in the cinnamon treated groups when compared to the untreated group. On the other hand, cyclin D1 was significantly (p<0.05) down-regulation in the Cinnamon treated groups when compared to the cancer control group.

**Conclusion**

The results of this study showed that Cinnamon has chemopreventive activity during oral carcinogenesis induced by 4-NQO.

**References**

[1] Valente VB, et al. Oral squamous cell carcinoma misdiagnosed as a denture-related traumatic ulcer: A clinical report. The Journal of Prosthetic Dentistry. 2016; 115(3):259-62.

[2] Ragin CC, Taioli E. Survival of squamous cell carcinoma of the head and neck in relation to human papillomavirus infection: Review and meta-analysis. International journal of cancer. 2007: 121(8):1813-20.

[3] Priyanga Ranasinghe, et al, Medicinal properties of ‘true’ cinnamon (Cinnamomum zeylanicum): a systematic review. 2013:[10.1186/1472-6882-13-275].

### OP03 Alterations of Blood-Brain Barrier Functions and Gene Expression following *Toxoplasma gondii* Infection

#### Mohammad Syamsul Reza Harun^1,2,^, Hany M. Elsheikha^2^ and Gareth Cave^3^

##### ^1^Advanced Medical & Dental Institute, Universiti Sains Malaysia, Penang, Malaysia; ^2^School of Veterinary Medicine & Science, University of Nottingham, United Kingdom; ^3^School of Science & Technology, Nottingham Trent University, United Kingdom

###### **Correspondence:** Mohammad Syamsul Reza Harun (mosyamsulre@usm.my)

**Background**

The apicomplexan protozoan parasite *Toxoplasma gondii* is the causative agent of toxoplasmosis, a zoonotic infection that affect roughly one-third of human population. A survey in 2017 among Malaysians’ pregnant women reported that 42.47% were seropositive with anti-*Toxoplasma* antibodies. *T. gondii* remarkable ability to disseminate via the bloodstream or within immune cells and to cross the blood-brain barrier was well reported. A better understanding of the mechanisms underpinning the parasite’s interaction with this barrier is crucial for the development of therapeutic interventions.

**Methodology**

The interactions of *T. gondii* with human brain microvascular endothelial cells (hBMECs) as blood brain barrier (BBB) model was evaluated using FITC-dextran flux-based permeability assay, transendothelial electrical resistance (TEER) measurements for tight junction integrity evaluation, viability, early/late apoptosis and cell cycle analyses using NC-3000™ image-based flow cytometry and tight junction genes (Occludin, PRKCA and ZO-1) and inflammatory genes (IL-6, P-GP and TNF-alpha) genes expression using RT-PCR. These experiments were performed at 3, 6, 24, 48 and 72 hours post infection (h.p.i) to represent early, middle and late stages of infection. This study then analysed the global transcriptomic changes in hBMECs caused by *T. gondii* infection in a time course study to uncover the underlying molecular mechanisms and signalling mechanisms that mediate the parasite-hBMECs interaction. Total RNA from *T. gondii* RH-infected hBMECs were obtained at 6, 24 and 48 h.p.i where mRNA and small RNA were sequenced using Illumina™ HiDeq 4000 and NextSeq 500 respectively. MRNA and small RNA sequencing reads were mapped to the annotated human GRCh37 and *T. gondii* ME 49 reference genomes before conducting differential expression analysis on uninfected versus infected cell reads. Genes expressed with false discovery rate (FDR) < 0.01 and >1 or <−1 log2 fold change were identified. Differently expressed genes were then validated with microarray and RT-PCR.

**Results and Discussion**

During the early and middle stages of infection, *T. gondii* maintained host cells’ viability, suppressed cell apoptosis by halting cells at the G0/G1 stage, induced up-regulation of IL-6 gene, reduced the permeability of infected cells compared to non-infected cells. Late stage of infection was marked by significant reduction of the cell integrity and vitality, and by significant increase of fragmented DNA. This study confirmed previous findings that *T. gondii* infection halts cell cycle progression and modulates the expression of cancer-like genes in infected cells. Integrated mRNA/miRNA expression profiling and pathway analysis showed multiple signalling pathways including, ATF4 activation, TP53 regulation, selenoamino acid metabolism and selenocysteine synthesis as key pathways regulated during *T. gondii* infection. MIRNA study identified increased expression of miR-7 and miR-17-92 family that possibly determine the fate of host cells in *T. gondii* infection. Also, *T. gondii’s* ribosomal proteins L36 and S12 were identified as key players in its pathogenesis and have good potential as vaccine candidates.

**Conclusion**

Our data provided new findings on *T. gondii* interaction with a 2D BBB model represented by hBMEC culture in a Transwell system, identified new genes and miRNAs altered by infection. These findings should facilitate further analysis of host-pathogen interactions in toxoplasmosis in preclinical animal models.

### OP04 The Effect of Black Rice (*Oryza sativa L.)* Extracts Administration on the Expression of VEGF Uterus and Fetal Weight in Rat (*Rattus norvegicus*) on Preeclampsia Model

#### Yudit Oktanella^1^, Meka L. Dwirinta^1^, Viski F. Hendrawan ^1^, Agung P. Warih^2^, Widjiati^3^

##### ^1^Department of Veterinary Reproduction, Faculty of Veterinary Medicine, Brawijaya University, Malang; ^2^Department of Biology, Faculty of Mathematics and Natural Sciences, Brawijaya University, Malang; ^3^ Department of Animal Reproduction, Faculty of Veterinary Medicine, University of Airlangga, Surabaya

###### **Correspondence:** Yudit Oktanella (yuditoktanella@gmail.com)

**Background**

Preeclampsia is one of the problems in obstetrics because it will increase both maternal and fetal morbidity and mortality. Preeclampsia is commonly recognized as pregnancy-induced hypertension or, in other words, acute hypertension on pregnancy, characterized by elevated blood pressure (hypertension) and protein found in the urine (proteinuria) and is associated with the dysfunction of placental and maternal blood circulation. The benefits of anthocyanin-rich vegetables and fruits consumption have been widely known to contain protective effect and antioxidant properties. Numerous studies have suggested that its antioxidant properties of anthocyanin were linked to their ability as an angiotensin-converting enzyme (ACE) inhibitor and reduce oxidative stress.

**Methodology**

In this study, animal models were induced with Triponyl sulphate at 70 mg/kg body weight in order to make a preeclampsia model rat. There were 5 groups in this study, namely: (CN) normal pregnant rat; (PC) mild-preeclamptic rat; (P1) preeclamptic rat treated with nifedipine; (P2) preeclamptic rat treated with anthocyanin, (P3) preeclamptic rat treated with a combination of nifedipine and anthocyanin.

**Results and Discussion**

The expression of VEGF showed a significant change with the average score between the groups on the cytoplasm of the endothelial cells. The fetal weight showed a significant difference with the average score between the groups, the highest one was the CN group with the average score 3.0±0.0 g. The analysis of inter variables showed those three doses of anthocyanin affected the VEGF variable positively for 5.8% and the bodyweight of fetus negatively for 19.5%.

**Conclusion**

Based on the result in this study, treating the preeclamptic rat using the combination of nifedipine and anthocyanin able to improve the VEGF expression in the uterus, but this effect was not well-correlated with the fetal weight. Further study is strongly recommended in order to evaluate any abnormality of the fetal development under anthocyanin treatment.

**References**

[1] Shen,Y., Jin, L., Xiao, P., Lu, Y and Bao, J. 2009. Total phenolics, flavonoids, antioxidant capacity in rice grain and their relations to grain color, size and weight. Journal of Cereal Science 49 (2009) 106–111

[2] Oktanella, Y., Firmawati, A., Aryani, DE and VF Hendrawan. 2017. Comparing Nifedipine, Anthocyanin, and Their Combination to Treat Animal Model of Preeclampsia: Effects on Serum Angiotensin II, Placental ICAM-1, Fetal and Placental weight. The 7th AIC-ICMR on Health and Life Sciences 2017, 375..

[3] Sutharut J, and Sudarat J. Total Anthocyanin Content and Antioxidant Activity Of Germinated Colored Rice. International Food Research Journal. 2012; 19(1): 215-221.

### OP05 Immuno-engineering and development of anti-m*KRAS* G12V single-chain variable fragment (scFv) fused-mHALT 1 recombinant immunotoxins against *KRAS*-positive colorectal cancer cells

#### Michelle Teo Yee Mun^1^, Hwang Jung Shan^2^, Adelene Song Ai Lian^3^, Renee Lim Lay Hong^1^ and Lionel In Lian Aun^1^

##### ^1^Faculty of Applied Sciences, UCSI University, Kuala Lumpur, Malaysia; ^2^Sunway Institute for Healthcare Development, Sunway University, Selangor, Malaysia; ^3^Faculty of Microbiology, University Putra Malaysia, Selangor, Malaysia

###### **Correspondence:** Lionel In Lian Aun (lionelin@ucsiuniversity.edu.my)

**Background**

*KRAS* oncogenes harbouring codon G12 substitutions are considered gate keeper mutations which drives oncogenesis in many cancers. To date, there are still no target specific vaccines or drugs available against this genotype, thus reinforcing the need towards the development of targeted therapies such as immunotoxins. Recently, new generation recombinant immunotoxins, which are toxin moieties fused to the single-chain variable fragment (scFv) portion of antibodies, have gained much attraction as cancer therapeutics following their ability to specifically target and eradicate abnormal cells without affecting non-targeted cells. This study aims to immuno-engineer and develop a recombinant anti-m*KRAS* scFv-fused mutant *Hydra* actonoporin-like-toxin-1 (mHALT 1) immunotoxin that is capable of recognizing and eradicating abnormal cells with a codon-12 mutated k-ras antigen.

**Methodology**

A G12V peptide mimotope (68-V) was designed to elicit antigen specific IgG titres against mutated K-ras antigen in immunized Balb/c mice. The RNA was extracted from splenocytes following ELISA confirmation on post-immunized mice sera and was reverse transcribed into cDNA. The anti-m*KRAS* G12V scFv library was constructed from a cDNA repertoire of variable regions of heavy chain (V_H_) and light chain (V_L_) fusions connected by a flexible glycine-serine linker, using splicing by overlap extension PCR (SOE-PCR). The anti-m*KRAS* G12V scFv fragments were constructed in pCANTAB5E phagemid and superinfected with M13K07 helper phage. Few rounds of bio-panning were performed to enrich high-affinity scFv clones against G12V control peptide. The binding specificity of anti-m*KRAS* G12V scFv protein was determined by phage- and monoclonal-ELISA. The anti-m*KRAS* G12V scFv fragment was fused to mHALT 1 toxin using SOE-PCR and cloned in pET22b vector. Expressed recombinant anti-m*KRAS* G12V scFv fused mHALT 1 immunotoxin was subjected to *in vitro* MTT cytotoxicity assay on various *KRAS* positive and *KRAS* negative cell lines.

**Results and Discussion**

Harvesting of splenocytes was carried out following ELISA confirmation of post-immunized mice sera with a significant elevation in G12V antigen specific-IgG (1). The V_H_ and V_L_ genes from spleen RNA of mice immunized with 68-V were amplified and randomly linked together, using SOE-PCR producing band sizes about 750 bp. Anti-m*KRAS* G12V scFv was constructed in pCANTAB5E phagemid vector with a library containing 1.8 x 10^7^ individual clones. After three rounds of bio-panning, the anti-m*KRAS* G12V-34 scFv antibody against G12V control mimotope was identified and confirmed without any cross-reactivity with other mimotopes or controls using ELISA. The anti-m*KRAS* G12V-34 scFv was fused to mHALT 1 toxin (2) using SOE-PCR, and was successfully expressed as inclusion bodies in *E. coli* (molecukar weight of ~46.8 kDa) and then refolded. HALT 1-scFv recombinant immunotoxin exhibited cytotoxicity effect on SW-480 colorectal cancer cells with IC_50_ of 30.17 μg/mL, with minimal cytotoxicity effect on normal human dermal fibroblast cells.

**Conclusion**

Anti-m*KRAS* G12V-34 fused mHALT 1 immunotoxin exhibited cytotoxicity effect on *KRAS* positive cancer cell. The development of such immunotoxins are potentially useful as a immunotherapeutic application against *KRAS*-positive malignancies. Additionally, this scFv can also be potentially developed as a serological based diagnostic tool for the detetcion of the G12V m*KRAS* genotype.

**References**

[1] Ng AWR, et al. In silico-guided sequence modifications of K-ras epitopes improve immunological outcome against G12V and G13D mutant *KRAS* antigens. PeerJ. 2018, 6:e5056.

[2] Yvonne J, et al. Mutagenesis and functional analysis of the pore-forming toxin HALT-1 from *Hydra magnipapillata*. Toxins. 2015, 7(2):407-422.

### OP07 PASD1: A potential biomarker in colorectal cancer?

#### Nur Fazilah Sukor^1^, Nur Maya Sabrina Tizen Laim^2^, Muaatamarulain Mustangin^2^, Azyani Yahaya^2^_,_ Ismail Sagap^3^, Rahman Jamal^1^ and Nor Adzimah Johdi^1^

##### ^1^UKM Medical Molecular Biology Institute, Universiti Kebangsaan Malaysia, Cheras, Kuala Lumpur, Malaysia; ^2^Department of Pathology, Faculty of Medicine, Universiti Kebangsaan Malaysia, Cheras, Kuala Lumpur, Malaysia; ^3^Department of Surgery, Faculty of Medicine, Universiti Kebangsaan Malaysia, Cheras, Kuala Lumpur, Malaysia

###### **Correspondence:** Nor Adzimah Johdi (adzimah@ppukm.ukm.edu.my)

**Background**

Per ARNT Sim Domain containing 1 (PASD1) protein is a cancer-testis antigen (CTA). It is not expressed in normal tissues but immunologically privileged sites such as normal testes tissue. However, it is widely expressed in some cancers. Its specificity makes PASD1 an attractive immunotherapeutic targets as DNA vaccine in cancers. Two PASD1 transcripts and proteins isoforms have been identified. PASD1_v1 transcript encodes 639 aa protein (PASD1a) and PASD1_v2 transcript encodes a longer 773 aa protein (PASD1b) due to alternative splicing event. Our study aims to investigate PASD1 mRNA and protein expression in colorectal cancer (CRC) and colorectal polyps (polyps) patients at UKMMC and correlate it with clinical data.

**Methodology**

Formalin-fixed paraffin-embedded (FFPE) tissue samples were collected from 30 CRC and 5 polyps patients. RNA was extracted using the RNeasy FFPE Kit (Qiagen), quantified using real-time PCR (qRT-PCR, Applied Biosystem) and analyzed. 3 primer sets used were primer A: 5’-TAC AGG AGC GGA AGA AGT GG-3’, primer B (5’-GCCCACAAACCTTGAAATCAC) and primer C (5’-AGCAGACCAGATTGATGCC). For PASD1 protein expression, the FFPE tissue sections were immunohistochemically stained using two PASD1 antibodies: 2ALCC136 (recognized both PASD1a and PASD1b proteins) and 2ALCC128 (recognized PASD1b protein only). Staining was detected using the EnVision™ FLEX Mini Kit (Dako).

**Results and Discussion**

9/15 (30%) of CRC samples showed significant PASD1 mRNA expression. This was 3 fold higher relative to the normal sample. No PASD1 mRNA expression was detected in polyp samples. PASD1 protein was moderately expressed in 12/30 (40%) CRC and 2/5 (40%) polyp samples using 2ALCC136 antibody. However, 2ALCC128 antibody staining were positive only in 2/30 (7%) CRC and none of the polyps samples. 6 CRC patients that were positive for both PASD1 mRNA and protein expressions, were in the later stage of cancer (Dukes’ C & D) and in the range 50-60 years old. This data suggests that PASD1 expression maybe a good biomarker an in CRC patients’ prognosis and disease progression.

**Conclusion**

PASD1 mRNA and protein level maybe a good indicator for CRC patients’ prognosis and disease progression, making it an attractive immunotherapeutic target CRC patients.

**References**

[1] Cooper, C. D., Liggins, A. P., Ait-Tahar, K., Banham, A. H., & Pulford, K. (2005). Protein Expression Profiles Confirm PASD1 as a Cancer Testis Antigen and a Potential Candidate for Lymphoma Immunotherapy. *Blood,* 106(11), 2825.

[2] Khan G, Denniss F, Mills KI, Pulford K, Guinn B. (2013)

PASD1: A Promising Target for the Immunotherapy of Hae

matological Malignancies. *J Genet Syndr Gene Ther*, 4: 186.

[3] Cooper, C. D., Liggins, A. P., Ait-Tahar, K., Roncador, G., Banham, A. H., & Pulford, K. (2006). PASD1, a DLBCL-associated cancer testis antigen and candidate for lymphoma immunotherapy. *Leukemia*, 20, pages 2172–2174.

### OP08 Development of aptamer thyroglobulin: new approach for autoimmune thyroid diseases (AITD) detection

#### Dyah Kinasih Wuragil^1,2^, Djoko Wahono Soeatmadji^3^ and Aulanni’am^4^

##### ^1^Doctoral Program of Medical Science, Faculty of Medicine, Brawijaya University, Malang, INDONESIA; ^2^Faculty of Veterinary Medicine, Brawijaya University, Malang, INDONESIA; ^3^Department of Internal Medicine, Faculty of Medicine, Brawijaya University, Malang, INDONESIA; ^4^Department of Chemistry, Faculty of Science, Brawijaya University, Malang, INDONESIA

###### **Correspondence:** Dyah Kinasih Wuragil (d_kinasih@ub.ac.id); Aulanni’am (aulani@ub.ac.id)

**Background**

Autoimmune thyroid diseases (AITD) is metabolite disorder caused by hereditary or environmental factors which characterized by the presence of inflammatory cell infiltration in thyroid gland. The large prevalence of this disease requires deeper studies in an effort to prevent the occurrence of AITD. Antigens that play a role in the development of this disease are thyroglobulin (Tg), thyroid peroxidase (TPO) and thyroid stimulating hormone receptor (TSHR) [1]. Previous study have developed and reported the preparation of experimental animal of thyroid disease using caprine thyroglobulin (cTg) in rats *(Rattus norvegicus)* [2,3]. Detection of AITD still relies on the identification of the function of thyroid gland and other indicators that cause tissue damage such as the presence of inflammation and protein identification. The function of the thyroid gland is mainly indicated by changes in hormone levels of Thyroxine (T4), and Tyroid stimulating hormone (TSH). The presence of Tg which reaches 75% in thyroid tissue has the potential to be autoantigen compared to TPO and TSHR. Thyroglobulin is a protein with a molecular weight of 660 kDa, it is necessary to modify the antigen with the large molecular weight to be used in detection schemes.

**Methodology**

DNA were isolated from Blood samples of AITD patients and normal people, then amplified the Tg encoding gene and compared the encoding gene sequences. The alignment is then performed on the sequence produced to determine the epitope that has high antigenicity. DNA library was determine with ssDNA with randomized design with conserved sequence as 5’-AGTAATACGACTCACTATAGGGAGTCGACCGACGAGAA-N40-TATGTGCGTCTACATCTAGACCTAT-3’ From this step, aptamer then was isolated using the Sequential Evolution of Ligands by Exponential Enrichment (SELEX) method. The resulting aptamer was then tested for immunogenicity to determine the presence of anti-thyroglobulin as a marker candidate in the detection kit with immunoassay approach.

**Results and Discussion**

The TG_F of 5’-GAGTACATTCTGGATCCTGG-3’ and TG_R of 5’-GGATCAGGTCACTAGTAGTG-3’ were performed amplification PCR product with molecular weight approximately 560 bp. This amplicon then was sequenced to analyze the highest antigenicity as a part in SELEX process. After aptamer was yielded, then it was coated in microwell plates to conduct ELISA in order to perform immunogenicity that can bind with anti-thyroglobulin that contains in sera.

**Conclusion**

Resulted aptamers in this research had immunogenic properties that can used to detect autoantibody of Tg to prevent AITD.

**References**

[1] Rassmusen, A.K., M.L. Hartoft-Nielsen, and U. Fieldt-Rasmussen. “Models to study the pathogenesis of thyroid autoimmunity”. Biochimie, 1999. 81: 511-515.

[2] Wuragil, D.K., D. A. Oktavianie, W. Riawan, A. Aulanni’am, and A.P.W. Marhendra.. “Rat induced thyroiditis for experimental animal models: comparative study of caprine thyroglobulin injection and sodium iodine (NaI) supplementation”. Journal of Animal and Veterinary Advances. 2014. Vol 13, No.20, pp: 1135-1138.

[3] Wuragil, D.K., N.M. Dliyaulhaq, A. Aulanni’am, and A.P.W. Marhendra. “Thyroid Stimulating Hormone (TSH) and Thyroxine Hormone (T4) Role in Thyroid Detriment on Hypothyroidsm Animal Models”. International Journal of Chem tech Research, 2015.Vol. 4 No.4, pp. 2092-2095.

### OP10 Application of a *Bm*SXP-specific recombinant monoclonal antibody in an antigen detection ELISA for Bancroftian filariasis

#### Anizah Rahumatullah^1^, Theam Soon Lim^2^, Hafiznur Yunus and Rahmah Noordin^1^

##### ^1^Institute for Research in Molecular Medicine (INFORMM), Universiti Sains Malaysia, Penang, Malaysia

###### **Correspondence:** Rahmah Noordin (rahmah@usm.my)

**Background**

Lymphatic filariasis (LF) is a debilitating mosquito-borne parasitic disease caused by three species of tissue/blood helminths namely *Wuchereria bancrofti*, *Brugia malayi* and *Brugia timori*. Of the three species, *W. bancrofti* infection (bancroftian filariasis) is responsible for 90% of the infections. LF is a neglected tropical disease associated with poverty in 53 countries [1]. The 2016 Global Burden of Disease reported that LF accounted for 189 million disability-adjusted life years [2]. In year 2000, the World Health Organization established the Global Programme to Eliminate Lymphatic Filariasis (GPELF), with the aim to eliminate LF as a public health problem by year 2020. The GPELF requires accurate diagnostic tools for various phases of its program. Diagnostic tests for detecting and/or quantifying circulating filarial antigens in humans have been developed for bancroftian filariasis but have been found to cross-react with *Loa-loa,* a non-lymphatic filarial parasite. In the present study, we have developed an alternative antigen-detection ELISA for bancroftian LF using our novel B*m*SXP-specific recombinant monoclonal antibody (5B) as the detecting antibody together with a polyclonal antibody against B*m*SXP as the capture antibody.

**Methodology**

Development of the antigen detection test comprised three main phases: i) Production and purification of anti-B*m*SXP rabbit polyclonal antibody. ii) Production of recombinant monoclonal antibody protein and conjugation with horseradish peroxidase (HRP). iii) The development of the assay to detect circulating *W. bancrofti* antigen in human serum samples. In total, the 5B-ELISA was performed using 124 samples comprising sera from 34 *W. bancrofti* microfilaria positive (mf+) individuals, 50 healthy individuals, and 40 individuals with other infections. In addition, the assay sensitivity was validated using spiked dried blood spots.

**Results and Discussion**

The results were used to plot a Receiver Operating Characteristic (ROC) curve and distribution graph. The 5B-ELISA showed a diagnostic sensitivity of 100% (95% CI: 88.4-100.0%) and diagnostic specificity of 100% (95% CI: 92.1-100.0%). This shows that the developed assay was able to detect all *W. bancrofti* microfilaremic individuals and did not cross-react with sera of people who were healthy and with other infection. In addition, the ELISA was also able to detect *W. bancrofti* antigen in the spiked dried blood spots samples, albeit with reduced OD values.

**Conclusion**

The developed assay using the novel 5B recombinant monoclonal antibody could potentially be a promising alternative antigen detection test for bancroftian filariasis. Further studies should be performed using a bigger sample size, including samples from non-microfilaraemic individuals from endemic areas, post-treatment patients, and *Loa-loa* infections.

**References**

[1] WHO 2017. Global programme to eliminate lymphatic filariasis: progress report,. Wkly Epidemiol Rec. 2016, 92: 594-607.

[2] DALYs GBD, Collaborators H, 2017. Global, regional, and national disability-adjusted life-years (DALYs) for 333 diseases and injuries and healthy life expectancy (HALE) for 195 countries and territories, 1990-2016: a systematic analysis for the Global Burden of Disease Study. Lancet. 2016, 390: 1260-1344.

### OP11 Gut microbiome secretome revealed possible microbial communication network failure in colorectal cancer

#### Siok-Fong Chin^1^, Putri Intan Hafizah Megat Mohd Azlan^1^, Luqman Mazlan^2^, Hui-min Neoh^1^, Raja Affendi Raja Ali^3^ and Rahman Jamal^1^

##### ^1^UKM Medical Molecular Biology Institute, Universiti Kebangsaan Malaysia, Kuala Lumpur, Malaysia; ^2^Department of Surgery, Faculty of Medicine, Universiti Kebangsaan Malaysia, Kuala Lumpur, Malaysia; ^3^Department of Medicine, Faculty of Medicine, Universiti Kebangsaan Malaysia, Kuala Lumpur, Malaysia.

###### **Correspondence:** Siok-Fong Chin (chinsiokfong@ppukm.ukm.edu.my)

**Background**

In recent decades, the importance of gut microbiota in maintaining human gut health and in the development of diseases has been extensively investigated. Alterations in microbial composition have been consistently reported in patients with CRC, including in our pioneer study on gut microbiome profiling of Malaysian CRC patients (TRGS/2/2014/UKM/02/3/1). Several bacterial candidates were found enriched in tumor tissues [1]. However, it is still unknown on how these gut bacteria may functionally contribute to CRC. The complex communication network between the host and microbes possibly achieved via protein secretions may have a role in exacerbating CRC. Therefore, we aim to profile the secretomic landscape of gut microbiome in patients with CRC-stricken gut by assessing the secretome in stool samples.

**Methodology**

A pilot study was carried out at the UKM Medical Centre, Kuala Lumpur. Patients were identified during the colonoscopy procedures and recruited post-histology confirmation. Stool samples from 26 clinically-diagnosed patients with CRC and 20 non-CRC control individuals were collected, homogenized and filtered followed by protein extraction and profiling by quantitative label-free proteomics using Nano-Liquid Chromatography TripleTOF Mass Spectrometry. The mass spectrum datasets were searched using MaxQuant against the microbial UniProt Fasta database. Statistical analyses were performed using the Statistical Package for Social Sciences (SPSS) version 22. Functional and integrative analyses of the identified proteins were performed using the Database for Annotation, Visualisation and Integrated Discovery (DAVID) [2] and MetaboAnalyst 4.0 [3] online tools, respectively.

**Results and Discussion**

Over 3700 proteins were identified from the gut microbes with nearly 3000 proteins were mapped to bacteria. We observed significant reduction of the microbial proteins in CRC, particularly from bacteria and fungi domains as compared to the non-CRC control. The ratio of Firmicutes to Bacteroides proteins has dropped remarkably by 83.3% in CRC. Proteins from wide-range genera were mostly undetected in CRC, either below detection or totally non-secreted. Interestingly, almost all the proteins identified in the CRC were not the same as those from non-CRC. The dissimilarities of the bacterial proteins run across the major genera whereby same organism identified in both groups showed different secretion profile. Functional annotation analysis on the identified proteins revealed none of the microbial proteins in CRC were of known specific function whereas the top non-CRC proteins are commonly involved in normal cell maintenance.

**Conclusion**

The secretome data from this study suggest possible disruption of microbial communication network in CRC. The absence of many maintenance-based important proteins may impact on the host-microbe interaction, resulted in the loss of natural microbial protection and thus pathological condition leading to CRC.

**References**

[1] Osman MA, et al. Aberrant gut mucosal microbiome signatures of Malaysian colorectal cancer patients. Gut. 2018, 67(Suppl 2): A11.

[2] Dennis JG, et al. DAVID: Database for Annotation, Visualization, and Integrated Discovery. Genome Biol. 2003; 4(5):3.

[3] Chong J, et al. MetaboAnalyst 4.0: towards more transparent and integrative metabolomics analysis. Nucl. Acids. Res. 2018; (doi:10.1093/nar/gky310).

### OP12 Cytotoxic and Antiangiogenic Properties of *Mentha Longifolia,* Grown in Saudi Arabia

#### Ibrahim Al-deeb^1,2^, Qasem Abdallah^3^, Amin Malik Shah Abdul Majid^2^, Yam Mun Fei^2^, Nozlena Abdul Samad^1^

##### ^1^Integrative Medicine Cluster, Institut Perubatan dan Pergigian Termaju (IPPT), Sains@BERTAM, Universiti Sains Malaysia, 13200 Kepala Batas, Pulau Pinang, Malaysia; ^2^Department of Pharmacology, School of Pharmaceutical Sciences, University Sains Malaysia, Minden, Penang 11800, Malaysia; ^3^Department of Pharmacology and Toxicology, College of Pharmacy, Taif University, Taif, Makkah 21974, Saudi Arabia

###### **Correspondence:** Nozlena Abdul Samad (nozlena@usm.my)

**Background**

Medicinal plants are well known to have a potential effect in mitigating the harmful adverse effects of conventional treatment. Recent years have seen the advent of a new generation of agents that directly target either the malignant cell itself or cells supporting tumor growth. Strategies in targeting tumor angiogenesis have been a crucial focus of intense research following observations that the growth and metastatic spread of tumors are dependent on their development of a vascular supply Therefore in this research, we investigated the cytotoxic and antiangiogenic properties of *Mentha Longifolia*, Lamiaceae family that is grown and cultivated in Saudi Arabia.

**Methodology**

Four types of extracts (WE, ME, 70EE and 100EE) were prepared from freshly harvested *Mentha Longifolia* leaves. The cytotoxicity of these extracts were investigated using MTT assay on selected cell lines (human colon cancer cell lines HT29 and HCT116, breast cancer cell line MDA-MB-231 and endothelial cell line EAhy926). The ex vivo studies were evaluated by Rat Aorta Ring Assay.

**Results and Discussion**

Water extracts (WE) do not show any significant cytotoxicity against all cell lines, while other extracts showed different IC50 against the cell lines. Interestingly, none of the extracts inhibit the growth of MDA-MB-231 and EAhy926. Their cytotoxicity are selective towards HT29 and HCT116. The IC50 values of the tree extracts on HT29 are 17±5, 30±3 and 33±3 μg/ml respectively. HCT116 were more resistant to the three extracts with IC50 values is double the IC50 value on the HT29. 100 μg/ml of each extract displayed anti-angiogenesis properties by the percentage value of the microvessels formation inhibition by rat aorta model are 61±2% (70EE), 51±3%(ME), 51%±5(100EE) and 22±1%(WE) respectively.

**Conclusion**

70% ethanolic extract (EE) *of Mentha Longifolia* showed selective activity against the growth of HT29 colon cancer cell line with the highest antiangiogenic properties among other extracts. Further studies are required to evaluate the molecular mechanism of cytotoxicity and antiangiogenesis properties which portray the important pathways in the pharmacological effects of these plants.

**References**

[1] Fidler MM, Soerjomataram I, Bray F. A global view on cancer incidence and national levels of the human development index. Int J cancer. 2016;139(11):2436-2446.

[2] Shankar E, Goel A, Gupta K, Gupta S. Plant flavone apigenin: an emerging anticancer agent. Curr Pharmacol reports. 2017;3(6):423-446.

[3] Banudevi S, Swaminathan S, Maheswari KU. Pleiotropic role of dietary phytochemicals in cancer: emerging perspectives for combinational therapy. Nutr Cancer. 2015;67(7):1021-1048.

### OP13 Enhanced production of a short peptide tagged recombinant Ss3a-7K protein as a potential biomarker for strongyloidiasis

#### Nur Hassanah Mohd Hassan^1^, Norsyahida Arifin^1^ and Rahmah Noordin^1^

##### ^1^Institute for Research in Molecular Medicine (INFORMM), Universiti Sains Malaysia, Penang, Malaysia

*BMC Proceedings* 2019, **Vol(Suppl no.):** P1

###### **Correspondence:** Rahmah Noordin (rahmah8485@gmail.com)

**Background**

Strongyloidiasis is a mysterious yet important parasitic disease that is hard to diagnose where approximately 100-370 million people are infected worldwide[1]. Human infection occurs when the infective filariform *Strongyloides stercoralis* larvae in contaminated soil penetrate the intact skin of feet sole or hand palm percutaneously through direct contact[2] and therefore leading to multiple silent complications especially among immunosupressed patients [3]. While microscopic examination remains a gold standard method, improved diagnosis is achieved through confirmatory assay with serological and/or molecular diagnostic approaches. In the current serodiagnosis of strongyloidiasis, recombinant proteins have been adopted in place of the use of native parasite antigens, although the availability of diagnostically potential proteins is still limited.

**Methodology**

The DNA sequence of Ss3a variant was custom-synthesized and sub-cloned into apET32 expression vector where seven peptide sequences of lysine (-7K) were added at the N-terminus of the original Ss3a sequence. The expression and purification of the recombinant protein were optimized and finally tested with 40 different human serum samples as a preliminary evaluation of its diagnostic potential.

**Results and Discussion**

Ss3a-7K protein was confirmed to be a soluble protein, hence the expression and purification processes that follow were based on a standard affinitive chromatography protocol for soluble proteins. The newly produced recombinant protein displayed a cleaner profile with enhanced yield and purity when compared to the original non-optimized Ss3a protein. Finally, the antigenicity of this recombinant protein was determined by western blot immunoassay was proven to be 80% (n=20) sensitive and 85% (n=20) specific.

**Conclusion**

Ss3a-7K is a new variant of Ss3a recombinant protein that is attached to a series of protein tags namely short amino peptide,

thioredoxin and histidine tags to enhance the solubility of the protein. In this study, we have successfully enhanced the yield and purity of Ss3a-7K recombinant protein, with significant diagnostic value. Hence, this protein merits further evaluation as a potential diagnostic biomarker for diagnosis of human strongyloidiasis.

**References**

[1] D. Buonfrate, F. Formenti, F. Perandin, and Z. Bisoffi, “Novel approaches to the diagnosis of Strongyloides stercoralis infection,” *Clin. Microbiol. Infect.*, vol. 21, no. 6, pp. 543–552, 2015.

[2] R. Varatharajalu and R. Kakuturu, “Strongyloides stercoralis: current perspectives,” *Reports Parasitol.*, no. May, p. 23, 2016.

[3] I. Reddy and G. Swarnalata, “Fatal disseminated strongyloidiasis in patients on immunosuppressive therapy: Report of two cases,” *Indian J. Dermatol. Venereol. Leprol.*, vol. 71, no. 1, p. 38, 2009.

### OP15 Expression of inflammatory mediators during in vitro macrophages infection by *Shigella flexneri* 2a

#### Nor Raihan Mohammad Shabani^1,2^, Chiuan Herng Leow^1^, Kirnpal Kaur Banga Singh^3^, Che Muhammad Khairul Hisyam Ismail^4^, Chiuan Yee Leow ^4^

##### ^1^Institute for Research in Molecular Medicine (INFORMM), Universiti Sains Malaysia, Penang, Malaysia; ^2^Faculty of Health Sciences, Universiti Teknologi MARA, Cawangan Pulau Pinang, Kampus Bertam, Penang, Malaysia; ^3^School of Medical Sciences, Universiti Sains Malaysia, Health Campus, Kubang Kerian, Kelantan, Malaysia; ^4^INFORMM, Universiti Sains Malaysia, Health Campus, Kubang Kerian, Kelantan, Malaysia

###### **Correspondence:** Chiuan Yee Leow (yee.leow@usm.my)

**Background**

*Shigella*, an intracellular Gram-negative bacteria that responsible for bacterial dysentery or shigellosis. The hallmark of this infection is the activation of the host immune cells to attack the invader. Macrophages play an important role in response to this intracellular pathogen. The mechanism by which *Shigella* infection regulates pro- or anti-inflammatory mediators during intestinal inflammation is still obscure. The investigation of both is crucial in determining the effectiveness of the immune system in providing protection against the intracellular bacterial infection. The purpose of this study is to identify the role of human macrophages in the control of infections caused by *Shigella flexneri* 2a (*S. flexneri* 2a).

**Methodology**

*S. flexneri* 2a mild (SH062) and virulence (SH057) strains were used to infect THP-1 monocytes-derived macrophages for 0, 6, 12 and 24 hr. Six target genes (TNFα, IL-1β, IL-6, IL-12, IL-10, and *inducible nitric oxide synthase (iNOS))* was determined by using real-time quantitative polymerase chain reaction (RT-qPCR). RT-qPCR was run for 40 cycles and the cycle threshold (Ct) values obtained from each sample was against GAPDH. Nitric oxide (NO) was determined by using NO assay kit E-BC-K036 (Elabscience, USA) on the supernatant of THP-1 derived macrophages coincubated with *S. flexneri* 2a for 0, 6, 12 and 24 h. Statistical analysis was performed by two-way ANOVA.

**Results and Discussion**

Inflammation caused by this disease is characterized by a balanced action between the pro- and anti-inflammatory cytokines. The expression of all pro-inflammatory mediators showed a marked increase in a time-dependent manner. Induction of TNFα, IL-1β, IL-6, IL-12, iNOS, and NO proved a pro-inflammatory response of the THP-1 derived macrophages towards *S. flexneri* 2a. This indicates that the human macrophages get involved in inflammation caused by *S. flexneri* 2a through the cytokines and NO production. SH057 was seen to be more powerful in eliciting the pro-inflammatory response than SH062. TNFα and IL-1β are the main chemotactic mediators involved in the recruitment of neutrophil to the inflammation sites. The production of IL-6 characterized as a factor that enhances antibody production in a B cell line. The IL-12 production leads to the activation of cell-mediated immunity through the stimulation of T helper 1 (Th1) cells. The induction of NO which is toxic to the *Shigella* indicates that macrophages have evolved defense mechanisms to minimize the possibility of becoming the host to this intracellular bacteria. IL-10 which is the anti-inflammatory cytokine was also induced by SH057 but to a much lesser level compared to the pro-inflammatory cytokines. This suggested an initial up-regulation of pro-inflammatory cytokines which could activate the immune response to fight against the bacteria followed with the up-regulation of anti-inflammatory cytokine.

**Conclusion**

In conclusion, the present finding showed that *S. flexneri* 2a causes THP-1 monocytes-derived macrophages to produce a pro-inflammatory response at the initial infection to attack the invader while later give the anti-inflammatory reaction to control the inflammation in response to the pro-inflammatory mediators. Overall, *S. flexneri* 2a has an immunomodulatory role in inducing a protective immune response in a model of human macrophages.

**Reference**

[1] Kazi A, Chuah C, Majeed ABA, Leow CH, Lim BH, Leow CY: Current progress of immunoinformatics approach harnessed for cellular- and antibody-dependent vaccine design. *Pathog Glob Health* 2018, 112:123-131.

### OP16 The Effect of Ionic Silver Water (Ag+) toward IL-10 and IL-1β Expression and Collagen Density in Incision Wound Healing Rats (*Rattus norvegicus*) Model

#### Wawid Purwatingsih^1^, Chanif Mahdi^2^, Aulanni’am Aulanni’am^2^, Patricia Erwini Easter Pusung^1^, Talitha Nindya Kirana^1^, Dhita Evi Aryani^3^ and Yudit Oktanella^4^

##### ^1^Department of Veterinary Biochemistry, Faculty of Veterinary Medicine, Brawijaya University, Malang, Indonesia; ^2^Department of Chemistry, Faculty of Sciences, Brawijaya University, Malang, Indonesia; ^3^Faculty of Pharmacy, University of Jember, Indonesia; ^4^Department of Veterinary Reproduction, Faculty of Veterinary Medicine, Brawijaya University, Malang, Indonesia

###### **Correspondence:** Wawid Purwatingsih (wawid_purwati@yahoo.com)

**Background**

A surgical wound or incision wound is a wound caused by sharp-edged objects such as a scalpel. The wound healing process is carried out by the stages of hemostasis, inflammation, proliferation and re-epithelization. Several studies indicate that onic silver water therapy has antibacterial and anti-inflammatory effects so that it can accelerate the inflammatory phase. This study aims to determine the effect of topically treatment of ionic silver water to the expression of IL-10, IL-1β and collagen density in incision wound model in rat model animals (*Rattus norvegicus*).

**Methodology**

This research was an experimental study using a completely randomized design (CRD). Twenty animal model of male Rattus norvegicus strain Wistar about 8-10 weeks old, body weight ranging from 179 - 205 g, later was divided into 4 treatment groups. Negative control group (normal group without incisions and treatment), P1 group (incisions with treatment of povidone iodine 10% 0.5 mL), P2 group (incisions with treatment of 0.5 mL ionic iilver Water (Ag+) Solution), and P3 group (incisions with combination treatment of Povidone iodine 10% with 0.5 mL ionic Silver Water (Ag+) Solution). IL-10 expression was examined using the flowcytometry and IL-1β expression was examined using immunohistochemistry method in One-Way ANOVA statistical analysis, and tukey test with 95% confidence level. Collagen density was seen by staining Masson’s Trichrome.

**Results and Discussion**

The results showed that single treatment of 20ppm ionic Silver Water (Ag+) solution (P2 group) significantly elevate the level of IL-10 expression (p<0.05) and the combination treatment of ionic Silver Water (Ag+) solution with Povidone iodine 10% (P3 group) was able to induce the best collagen growth. IL-1β expression showed a relatively significant reduction on P3 group. The occurrence of the wound healing process is inseparable from the role of growth factors and cytokines. Cytokines are proteins that function as inflammatory mediators and regulators of immunity (Velnar, 2013). One cytokine that plays an important role in this process is Interleukin-10 (IL-10). IL-10 is one of the anti-inflammatory cytokines that functions to inhibit the production of several other types of cytokines namely pro-inflammatory cytokines such as IL-1, TNFα, IL-1β and IL-12. Ag (Silver) with an antibacterial and anti-inflammatory effect (Knetsch and Koole, 2011) helps to speed up the healing process of wounds. Ionic silver water (Ag+) suppresses the level of inflammatory markers such as inflammatory cells so that this process can be completed and enter the proliferation phase.

**Conclusion**

Based on the result of this study, it could be concluded that ionic Silver Water (Ag+) solution can accelerate incisional wound healing in rats (*Rattus norvegicus*) by increasing the level of IL-10 expression and collagen growth and suppressing the IL-1β expression.

**References**

[1] Knetsch M. L., Koole L. H. 2011. New Strategies In the Development of Antimicrobial Coatings: The Example of Increasing Usage of Silver and Silver Nanoparticles. Polymers.

[2] Velnar, T. 2013. The Wound Healing Process: an Overview of the Cellular and Molecular Mechanisms. Journal of International Medical Research. 37: 1528.

### OP17 DNA Aptamer Possesses G/C-rich Binds Specifically to Recombinant Human ICAM-1

#### Nik Abdul Aziz, Nik Kamarudin^1^, Nurfadhlina, Musa^2^, Nur Fatihah, Mohd Zaidi^1^ and Khairul Mohd Fadzli, Mustaffa^1^

##### ^1^ Institute for Research in Molecular Medicine (INFORMM), Health Campus, Universiti Sains Malaysia, Malaysia; ^2^ School of Medical Sciences, Health Campus, Universiti Sains, Malaysia.

###### **Correspondence:** Khairul Mohd Fadzli, Mustaffa (khairulmf@usm.my)

**Background**

*Plasmodium falciparum* was known as the most common parasite strain associated with cerebral malaria when high burden parasites were observed in a brain microvasculature blood vessel. Parasite cytoadherence to microvasculature is mediated by intercellular adhesion molecule-1 (ICAM-1) which can be upregulated by TNF-α during the infection [1]. The aptamer is a single-stranded nucleic acid (DNA or RNA) that have the capability to bind to any target molecules at high specificity and affinity like an antibody [2]. Thus, this study was aimed to isolate the DNA aptamer against ICAM-1 which potentially could be used as anti-cytoadhesion of infected erythrocyte to ICAM-1 and prevent cerebral malaria.

**Methodology**

The DNA aptamer selection was conducted using 83-mers nucleotide libraries against recombinant human ICAM-1 coupled Protein A Dynabeads (1X PBS, pH 7.4; 1.5 mM of MgCl_2_; 1% BSA]. The aptamer from the 8^th^ cycles was cloned and sequenced. The aptamer structure analysis was done using online mfold software. The binding analysis of aptamer was performed using ELONA assay. The specificity of isolated aptamers was evaluated against BSA and recombinant human CD36. The aptamer competitive binding assay was performed using commercialized monoclonal antibody 15.2.

**Results and Discussion**

Isolation of DNA aptamer against ICAM-1 was successfully achieved after 8^th^ round of SELEX. The sequencing analysis of twenty-five isolated aptamers candidate was clustered into twenty-three groups based on their sequence similarity and was also grouped to ‘T-rich’, ‘T/G-rich’, and ‘G-rich’ families. We found that there were seven aptamers candidates showed significant binding to ICAM-1 compared to DNA library. The aptamer that possesses G/C-rich sequence (DI05) was showed has the highest binding ability compared to the T-rich family (DI20 and DI33) followed by G-rich family (DI31). The balanced mixture of T/G/C bases within clone DI05 was thought might enhance the conformation tertiary structure of aptamer. The binding specificity was showed that DI05 aptamer significantly had no cross-reactivity to BSA and CD36. Finally, the competitive binding assay was showed significant reduction of absorbances for DI05, DI20, DI31 and DI33 in the presence of mAb 15.2. The reduction of absorbance may have been caused by the competitive inhibition of mAb 15.2 at the same epitope site of DNA aptamer binding to ICAM-1.

**Conclusion**

This study was successfully isolated DNA aptamer (DI05) which could potentially be used as adjunct therapy targeting cerebral malaria.

**References**

[1] Dunst J, Kamena F, Matuschewski K: Cytokines and Chemokines in Cerebral Malaria Pathogenesis. Frontiers in Cellular and Infection Microbiology 2017, 7(324).

[2] Nik Kamarudin N, Mohammed N, Mustaffa K: Aptamer Technology: Adjunct Therapy for Malaria. Biomedicines 2017, 5(1):1.

### OP18 Characterization of an outer-membrane proteins as potential cellular- and antibody-dependent vaccine candidates against *Shigella flexneri*

#### Che Muhammad Khairul Hisyam Ismail^1^, Ada Kazi^1^, Candy Chuah^3^, Boon Huat Lim^2^, Chiuan Herng Leow^4^, Kirnpal Kaur Banga Singh^3^, Chiuan Yee Leow^1^, Khairul Mohd Fadzli Mustaffa^1^, Amy Amilda Anthony^1^

##### ^1^Institute for Research in Molecular Medicine (INFORMM), Universiti Sains Malaysia, Kelantan, Malaysia; ^2^School of Health Sciences, Universiti Sains Malaysia, Kelantan, Malaysia; ^3^School of Medical Sciences, Universiti Sains Malaysia, Kelantan, Malaysia; ^4^Institute for Research in Molecular Medicine (INFORMM), Universiti Sains Malaysia, Penang, Malaysia.

###### **Correspondence:** Chiuan Yee Leow (yee.leow@usm.my); Che Muhammad Khairul Hisyam Ismail (chekhairul93@yahoo.com.my)

**Background**

Bacillary dysentery is one of the most common communicable diarrheal infections recently. There are approximately 169 million cases of shigellosis reported worldwide. The disease is transmitted by a group of Gram-negative intracellular enterobacteria known as *Shigella flexneri, S. sonnei, S. dysenteriae and S. boydii* [1]. Conventional treatment regimens for *Shigella* have been less effective due to the development of resistant strains against antibiotics. Therefore, an effective vaccine for the long term control of *Shigella* transmission is urgently needed [2].

**Methodology**

In this study, reverse vaccinology approach was employed to identify most conserved and immunogenic outer membrane proteins (OMPs) of *S. flexneri 2a*. A primary data was retrieved form Genbank based from two virulence strain from main pathogenic *Shigella* group; *S. boydii* CDC and *S. dysenteriae* SD197 for orthologous comparison. The most conserved and immunogenic OMPs of S. flexneri 2a which can be potent vaccine candidates were initially identified. The shortlisted proteins were then predicted for their antigenicity as potential epitopic sequences using validated online immunoinformatics software.

**Results and Discussion**

Five OMPs including fepA, ompC, nlpD_1, tolC and nlpD_2 were identified as potential vaccine candidates. Protein-protein interactions analysis using STRING software revealed that five of these OMPs may potentially interact with other intracellular proteins which are involved in beta-lactam resistance pathway. Each of these OMPs contains regions which are capable to induce B- and T-cell immune responses. Analysis acquired from this study showed that five selected OMPs have great potential for vaccine development against *Shigella* infection. The predicted immunogenic epitopes can also be used for development of peptide vaccines or multi-epitope vaccines against human shigellosis.

**Conclusion**

Utilization of bioinformatics tools in designing a potential vaccine candidates showing that this is a promising strategy for the future vaccine development against global persistent infection.

**References**

[1] Livio, S., et al., *Shigella isolates from the global enteric multicenter study inform vaccine development.* Clin. Infect. Dis. 2014, 59(7): p. 933-941.

[2] Singh, K.-K.B., et al., *A 9-year study of shigellosis in Northeast Malaysia: antimicrobial susceptibility and shifting species dominance.* J. Public Health, 2011, 19(3): p. 231-236.

### OP19 Anti-idiotypic suppression of embryo alpha-fetoprotein degradation

#### I.A. Franceva^2^, J.K. Ukibayev^1^, U.M. Datkhayev^1^, A.P. Francev ^2^, T.G. Goncharova^3^, D.A. Myrzakozha^1^

##### ^1^Kazakh National Medical University named after S.D. Asfendiyarov, Almaty, 050010, Kazakhstan; ^2^Research and production association "Fran", Almaty, 050010, Kazakhstan; ^3^Kazakh Research Institute of Oncology and Radiology, 050010, Kazakhstan

###### **Correspondence:** D.A. Myrzakozha (myrzakozha.d@kaznmu.kz)

**Background**

The synthesis of antibodies and cellular receptors for alpha-fetoprotein is carried out, respectively, B - and T-lymphocytes. The complement, as one of the main components of humoral immunity, is always present in the blood serum, and only anti-idiotypic antibodies from alpha-fetoprotein synthesized can be antibodies that interact with antibodies and cell receptors directed against embryo alpha-fetoprotein the female’s immune system, as the embrion does not have a functioning immune system. The interaction of anti-idiotypic antibodies against alpha-fetoprotein with antibodies and cell receptors on the surface of B- and T-lymphocytes against alpha-fetoprotein, in combination with complement, will completely destroy cell clones that can synthesize anti-bodies and cellular receptors, directed against embryo alpha-fetoprotein. The aim of this work is to detect in female blood anti-idiotypic antibodies from alpha-fetoprotein, which selectively suppress the synthesis of anti-bodies against embryo alpha-fetoprotein.

**Methodology**

The work used the blood of the woman in childbirth. The amount of 5 ml blood was placed in a thermostat at a temperature of 36 °C until a clot formed. The resulting blood clot was surrounded by a thin wire to separate it from the walls of the tube. The tube was carefully tilted to drain the serum. The resulting blood serum in an amount of 1.5 ml. applied to a pre-equilibrated starting pH, 20 mM sodium phosphate buffer pH 7.0, a miniature column filled with Sigma recombinant (*E. coli*) fast flow protein G Sepharose (Germany) capable of adsorbing human and goat IgG immunoglobulins only, to separate IgG antibodies from other components of the blood serum. The affinity chromatography of serum proteins on a protein G Sepharose column was carried out according to a conventional method. The elution profile was recorded using a 2151 UV Variable Wavelength Monitor flow densitometer (LKB, Sweden) at a wavelength of 280 nm. Fractions of serum proteins obtained on a Protein G Sepharose column were neutralized to pH 7.0 by the addition of a Tris base solution.

**Results and Discussion**

As a result of the work, the presence of anti-idiotypic antibodies from alpha-fetoprotein in the IgG fractions of serum immunoglobulins obtained after affinity chromatography on a protein G Sepharose column was determined by the enzyme immunoassay, by their ability to interact with antibodies against alpha-fetoprotein (idiotype - anti-idiotype interaction). The work was performed on an ABBOTT AxSYM enzyme-linked immunosorbent analyzer (USA), during which the presence of alpha-¬ fetoprotein in the amount of 0.21 ng / mL was detected in serum IgG fractions of immunoglobulins, although there, could not be a priori.

**Conclusion**

Thus, the presence of anti-idiotypic antibodies against alpha-fetoprotein in the blood of a female, which does not allow the development of antibodies against alpha-fetoprotein of the embryo and the fetus, has been shown.

**References**

[1] G.S. Vasilyeva, I.A. Frantseva et al. Studies on the effect of Normogen on the metastatic ability of an ovarian affinity tumor (AfOI) in rats with lung tissue tropism. Oncology and Radiology of Kazakhstan № 2 (11), 2005 p. 27 – 31.

[2] Joost P.M. Melis, Kristin Strumane et all. Complement in therapy and disease Regulating the complement system with antibody-based. Molecular Immunology 67, pp. 117–130, 2015.

[3] E.A. Lesovaya, A.P. Kaplun The therapy of cancer diseases by means of of targeted complement activation, Russian Biotherapeutic Journal No. 3, vol. 7, 2008. [3] First author, et al. Title of book chapter. In: First editor, et al. (eds). Book name. City, Publisher, Year; p. first page-last page.

### OP22 Isolation and Evaluation of RNA Aptamers Against 16 kDa *Mycobacterium tuberculosis* Antigen Protein

#### Nurul Adila Mohammed^1^; Nik Abdul Aziz Nik Kamarudin^1^; Basyirah Ghazali^1^; Judy Aisya Sat^1^; Khairul Mohd Fadzli Mustaffa^1^

##### ^1^Institute for Research in Molecular Medicine, Universiti Sains Malaysia, Health Campus, 16150 Kubang Kerian, Kelantan,Malaysia

Tel: ; Fax: +6097648673

*BMC Proceedings* 2019, **Vol (Suppl no.):**P1

###### **Correspondence:** Khairul Mohd Fadzli Mustaffa +6097672435 (khairulmf@usm.my)

**Background**

Tuberculosis (TB) is an airborne ancient disease that has affected mankind which is chronic caused by *Mycobacterium tuberculosis* (*M.tb.*). There were several laboratory of diagnosis available for TB screening such as smear microscopy, culture and GeneXpert® that already shown a reliable result to diagnose patients with active TB. However, all of the methods are not for detection of latent TB infection (LTBI). Although IGRAs (Interferon Gamma Release Assay) had been introduced for diagnosis of LTBI, unfortunately the test is expensive and laborious laboratory tests. Nonetheless, the test is not accessible for poor countries which is mostly having high burden of TB due to cost and have no fast point-of-care setting. Thus, a new rapid test urgently is needed to improve the recent diagnosis method for LTBI which is more affordable and easily implemented. To date, aptamers has been received considerable attention due to its recognition properties that could mimic function of antibody against the target with high affinity and specificity [1]. The 16 kDa of *Mycobacterium tuberculosis* was chosen as study prime candidate due to its importance in tuberculosis latency and immunodominant property [2].

**Methodology**

The RNA aptamers against 16 kDa antigen protein was generated using random pool RNA library through *in vitro* selection process (SELEX). The successful isolated aptamers were then clustered together according to their similar unique sequences. The secondary structure and motif sequence was identified using free online software. In this study, EMSA was performed using agarose gel electrophoresis to evaluate the binding and the dissociation constant (Kd) determination.

**Results and Discussion**

Six clusters of RNA aptamers were successfully isolated and its secondary structure was predicted with the lowest Gibbs free energy was chosen for each group. The motif sequence that had been discovered shown a correlation with the binding site of secondary structure which is single stranded and within the randomised region. The discovery of motif sequence also presumed that the aptamers had a biological function for aptamer-protein interaction. By measuring the band intensity of the gel profile, the dissociation constant was evaluated for each cluster. The aptamer (TB_APG01) had the highest frequency clones (14/104) within the clusters isolated after selection process and TB_APG04 had highest Kd value (3.935 μM).

**Conclusion**

As a conclusion, six isolated clusters of RNA aptamers were successfully analysed for their secondary structure and motif sequences. The analysis of binding between selected potential aptamers with 16 kDa antigen protein showed a significant interaction for each cluster. The aptamer TB_APG04 which was identified had the lowest Kd value which indicated the strongest aptamer against 16 kDa within the clusters.

**References**

[1] Khati, M. The future of aptamers in medicine. J. Clin. Pathol. 2010, 63(6), 480-487.

[2] Siddiqui, K. F., Amir, M. & Agrewala, J. N. Understanding the biology of 16 kDa antigen of Mycobacterium tuberculosis: scope in diagnosis, vaccine design and therapy. Crit. Rev. Microbiol. 2011, 37(4), 349-357.

### OP23 Mass Spectrometry analyses on *Entamoeba histolytica* membrane sub-proteome

#### Nurulhasanah Othman^1^, Jorim Ujang^1^, Asmahani Azira Abdu Sani^2^, Lim Boon Huat^3^, Rahmah Noordin^1^

##### ^1^Institute for Research in Molecular Medicine (INFORMM), Universiti Sains Malaysia, 11800, Penang Malaysia; ^2^Malaysia Genome Institute (MGI),43000, Selangor, Malaysia; ^3^School of Health Sciences, Universiti Sains Malaysia, 16150, Kelantan, Malaysia.

###### **Correspondence:** Nurulhasanah Othman (nurulhasanah@usm.my)

**Background**

*Entamoeba histolytica* is a protozoan parasite that causes amoebiasis. Infection of this parasite may lead to amoebic dysentery and amoebic liver abscess, which is fatal if left untreated. Until now very less studies were performed on the membrane proteome of *E. histolytica*. As membrane proteins could be very promising target for new drug and vaccine designs, therefore, proteomic analyses were performed to explore the membrane sub-proteomes of *E. histolytica* trophozoites in this study.

**Methodology**

The culture of *E. histolytica* trophozoites were performed according to Diamond et al., (1995). Then, the membrane fraction of the trophozoites were extracted using three methods: two commercial kits (ProteoExtract® from Calbiochem and ProteoPrep® from Sigma), and a conventional laboratory method. The membrane protein and cytosolic fractions from each method were tryptic-digested. Then, the digested peptides were analysed using LC-ESI-MS/MS and LC-MALDI-TOF/TOF for protein identification. The identified proteins were then analysed by a bioinformatic software namely TOPCONS 2.0 to predict the presence of transmembrane helix and/or signal peptide.

**Results and Discussion**

The results showed that the ProteoExtract® kit and the conventional method extracted higher protein yields compared to the ProteoPrep® kit. The combined data from LC-MALDI-TOF/TOF and LC-ESI-MS/MS identified 490, 492, and 587 proteins extracted using the ProteoExtract®, ProteoPrep®, and conventional methods, respectively. *In-silico* analysis predicted 109 (22%), 237 (48%) and 182 (31%) membrane proteins from the ProteoExtract®, ProteoPrep® and conventional method extracts, respectively. Furthermore, the identification of the cytosolic and membrane protein fractions showed that the ProteoPrep® extraction kit was the most selective and specific for the extraction of the membrane proteins.

**Conclusion**

In conclusion, the results revealed 249 *E. histolytica* membrane proteins. Furthermore, this study confirmed that the use of two types of mass spectrometers enhances proteome coverage. The data generated has increased the understanding on the types of proteins that reside at the parasite’s membrane. The identified proteins will be useful for further studies in understanding the pathogenesis of amoebiasis and the roles the proteins play in the host-parasite interactions.

**References**

[1] Diamond L.S., Clark CG, Cunnick C.C. YI-S, a casein-free mediumforaxeniccultivationofEntamoebahistolytica,related Entamoeba, Giardia intestinalis and Trichomonas vaginalis. 1995. J. Eukaryot. Microbiol. 42: 277–278.

### OP24 Development of Multiplex Real-time RT-PCR for Detection and Subtyping of Avian Influenza Virus

#### May Ye Yee^1^, Shaharum Shamsuddin^2^ and Ismail Aziah^1^

##### ^1^Institute for Research in Molecular Medicine (INFORMM), Universiti Sains Malaysia, Penang, Malaysia; ^2^School of Health Sciences, Health Campus, Universiti Sains Malaysia, 16150 Kubang Kerian, Kelantan, Malaysia

BMC Proceedings 2019, Vol(Suppl no.): P1

###### **Correspondence:** Ismail Aziah (aziahismail@usm.my)

**Background**

Avian influenza is an infectious disease in avian species caused by avian influenza virus (AIV). AIVs of subtype H5, H7 and H9 are the most prominent haemagglutinin (HA) subtypes that resulted in serious impacts in poultry industry and caused infection in human occasionally [1] [2]. Real-time reverse transcription (RT)-PCR (qRT-PCR) assays used currently do not include internal amplification control (IAC) to ensure proper PCR and eliminate false-negative results. This study aimed to develop duplex qRT-PCR assays for detection and identification of H5, H7 and H9 subtypes of AIV with inclusion of IAC.

**Methodology**

Primers and probes specific for the matrix (M) gene for detection of AIV, H5-, H7 and H9-HA gene for subtyping were designed and validated with 4 reference viruses. IAC utilized a synthetic plasmid DNA harboring human glyceraldehyde 3-phosphate dehydrogenase (GAPDH) amplified by specifically designed primers and probe. IAC interference to the amplification of target gene in duplex reaction was initially evaluated to determine the optimal quantity of IAC template. The performance of duplex qRT-PCR assays using the optimized reaction conditions was then evaluated with respect to corresponding simplex reaction.

**Result and Discussion**

Primer and probe of M gene detection were tested positive with all 4 viruses. No cross-reactivity was detected for primer-probe sets of subtyping, indicating high specificity of the assays. However, the assays need to be tested with more avian pathogens to check for diagnostic specificity. Interference of IAC was absent except for H9-HA assay. Further optimization on concentration of primer and probe is required to improve the analytical sensitivity. All duplex assays with addition of 102 copies IAC were successfully developed except for H9-HA assay. The quantification cycle (Cq) value difference between the simplex and duplex assay was within 1Cq and the amplification efficiency remained in the accepted range. The detection limit of all duplex assays except H9-HA was 10 copies of recombinant plasmid containing target gene per reaction with linear dynamic range of 10 to 107 copies. Reproducibility test and field samples evaluation are required to provide more evidence of feasibility for future use.

**Conclusion**

Rapid detection and subtyping of AIV are crucial for immediate control actions. Incorporation of IAC in qRT-PCR assay could help to avoid false-negative results reporting. Furthermore, triplex assays that combines H9-HA with H5- or H7-HA detection could also be developed in order to save the cost of analysis.

**References**

[1] Tahir, M. S., Mehmood, D., Sultan, A. U., Saeed, M. H., Khan, A. R., Ansari, F., Salman, M. M. & Majeed, K. A. (2016). A Modified Strategy of Multiplex RT-PCR for Simultaneous Detection of H5, H7, and H9 Subtypes of Avian Influenza Virus Based on Common Forward Oligo. The Journal of Applied Poultry Research, 25(3), 322-327.

[2] Monne, I., Ormelli, S., Salviato, A., De Battisti, C., Bettini, F., Salomoni, A., Drago, A., Zecchin, B., Capua, I. & Cattoli, G. (2008). Development and Validation of A One-Step Real-Time PCR Assay for Simultaneous Detection of Subtype H5, H7, and H9 Avian Influenza Viruses. Journal of Clinical Microbiology, 46(5), 1769-73.

### OP25 *In silico* identification of miR-100 associated target genes and its potential molecular mechanisms in acute promyelocytic leukaemia (APL)

#### Emilia Apidi^1^, Wan Rohani Wan Taib^1^, Rosline Hassan^2^ and Imilia Ismail^1^

##### ^1^School of Biomedicine, Faculty of Health Science, Universiti Sultan Zainal Abidin, Gong Badak Campus, 21300 Kuala Terengganu, Malaysial; ^2^Department of Haematology, School of Medical Sciences, Universiti Sains Malaysia, Health Campus, 16150 Kubang Kerian, Kelantan, Malaysia.

###### **Correspondence:** Imilia Ismail (imilia@unisza.edu.my)

**Background**

Acute promyelocytic leukaemia (APL) is the highly treatable form of AML with the cure rate of nearly 90% and mortality under 10%. However, immediate attention should be given to the patients due to life-threatening coagulopathy. APL disease comprehension, particularly from the molecular perspective is imperative to improve disease outcome. MicroRNAs (MiRNAs) are non-coding RNAs (ncRNAs) that play fundamental roles in dictating cellular fates and disease development. Our previous study on miRNA expression profiling using the NanoString nCounter® platform found that miR-100 was the most up regulated miRNAs in newly diagnosed APL patients. The present study aims to (i) identify miR-100 associated target genes; (ii) to perform functional enrichment analysis of miR-100 identified target genes and (iii) to identify experimentally validated miR-100 target genes in human cancers so as to decipher the potential molecular mechanisms regulated by miR-100 in APL.

**Methodology**

MiRWalk2.0 online database was used to identify miR-100 target genes. STRING v11.0 and Cytoscape 3.6.0 software plug-in ‘Network Analyzer’ and ‘BINGO’ were applied for the construction of protein-protein interaction (PPI) network, identification of hub genes, Gene Ontology (GO) term enrichment analysis, KEGG and PANTHER pathway analysis. Finally, miRTarbase 7.0 was employed to search for experimentally validated miR-100 target genes in human cancers as documented from the previous studies.

**Results and Discussion**

A total of 101 target genes were detected. Analysis of the PPI network comprising of 101 nodes and 29 edges led to the identification of 5 hub genes, *MTOR*, *RPTOR*, *FOXO3*, *RAP1B* and *IGF1R* as potential biomarker genes and therapeutic targets. In addition, functional and signaling pathway analysis unveils various regulatory mechanisms and pathways associated with miR-100 target genes, thereby suggesting the complexity of cancer genesis. Data from miRTarbase 7.0 revealed several target genes for miR-100 have been validated in human cancers.

**Conclusion**

Considering that miR-100 was implicated in various regulatory mechanisms, cancer associated pathways and human cancers, miR-100 might play an important role in APL leukemogenesis, by targeting *MTOR*, *RPTOR*, *FOXO3*, *RAP1B* and *IGF1R*.

### OP26 Modulation of osteocyte biomarkers by annatto tocotrienol in protecting against metabolic syndrome-induced osteoporosis in rats

#### Sok Kuan Wong, Kok-Yong Chin, Soelaiman Ima-Nirwana

##### Department of Pharmacology, Faculty of Medicine, Universiti Kebangsaan Malaysia, Jalan Yaacob Latif, Bandar Tun Razak, 56000 Cheras, Kuala Lumpur, Malaysia

###### **Correspondence:** Soelaiman Ima-Nirwana (imasoel@ppukm.ukm.edu.my)

**Background**

Annatto tocotrienol has been previously shown to exert skeletal-promoting effects mainly through the regulation of bone formation and resorption activities governed by osteoblasts and osteoclasts [1]. The role of tocotrienol on osteocytes has not been explored. This study aimed to investigate the effects of annatto tocotrienol in the modulation of osteocyte-mediated bone remodeling in an osteoporotic rat model induced by metabolic syndrome.

**Methodology**

Thirty male Wistar rats were purchased and divided randomly into five groups (n=6/group). The baseline group was sacrificed upon arrival. The normal group was fed with standard rat chow and tap water. The other three groups were given high-carbohydrate high-fat (HCHF) diet and 25% fructose water. Food and drinks were provided *ad libitum* throughout the study period. After 8 weeks, the three groups of HCHF animals were orally administered with tocopherol-stripped corn oil, 60 and 100 mg/kg annatto tocotrienol, respectively. At week 20, all the animals were sacrificed. Right tibia was harvested, cleaned of soft tissues and homogenised. Protein was extracted from the metaphyseal region of proximal tibia for the assessment of osteocyte biomarkers [osteoprotegerin (OPG), soluble receptor activator of nuclear factor-kappa B ligand (sRANKL), sclerostin (SOST), Dickkopf-related protein 1 (DKK-1), fibroblast growth factor-23 (FGF-23) and parathyroid hormone (PTH)].

**Results and Discussion**

The rats fed with HCHF diet had higher sRANKL, SOST, DKK-1, FGF-23 and PTH (p<0.05). Treatment with 60 and 100 mg/kg annatto tocotrienol reversed the elevated sRANKL and FGF-23 in the HCHF animals (p<0.05). Supplementation with 100 mg/kg annatto tocotrienol resulted in the reduction in SOST and DKK-1 in the HCHF animals compared to untreated animals (p<0.05). OPG and RANKL are important regulators for osteoclastogenesis, whereby a high RANKL:OPG ratio favors osteoclast formation and bone resorption [2]. High levels of SOST and DKK-1 antagonize wingless (Wnt) signaling thereby inhibiting osteoblastogenesis and bone formation [2]. FGF-23 and PTH regulate phosphate homeostasis and vitamin D metabolism, suggesting their role in bone metabolism [3].

**Conclusion**

Annatto tocotrienol alters expression of osteocyte-related proteins in favor of bone formation in rats with metabolic syndrome. These changes partly contribute to the bone protective effects of annatto tocotrienol in metabolic syndrome and need to be validated in further studies.

**References**

[1] Wong SK, et al. Exploring the potential of tocotrienol from Bixa orellana as a single agent targeting metabolic syndrome and bone loss. Bone. 2018, 116:8-21.

[2] Bellido T. Osteocyte-driven bone remodeling. Calcif. Tissue. Int. 2014, 94:25-34.

[3] Jüppner H. Phosphate and FGF-23. Kidney Int. 2011, 79:S24-S27.

### OP28 Effects of Annatto Tocotrienol Formulated with Self-Emulsifying Drug Delivery System on Bone Microstructure in a Rat Model of Postmenopausal Osteoporosis

#### Nur Vaizura Mohamad, Ima Nirwana Soelaiman, Chin Kok Yong

##### Department of Pharmacology, UKMMC, Jalan Yaacob Latif, Bandar Tun Razak, Cheras 56000, Kuala Lumpur Universiti Kebangsaan Malaysia Medical Centre, Kuala Lumpur, Malaysia

###### **Correspondence:** Chin Kok Yong (chinkokyong@ppukm.ukm.edu.my)

**Background**

Postmenopausal women are particularly susceptible to osteoporosis due to the abrupt cessation of oestrogen production [1]. Annatto tocotrienol (AnTT) has been shown in previous study to prevent against osteoporosis [2] but its application is plagued by two main issues. Firstly, there is no evidence that it can reverse bone loss in a model of established. Secondly, the unformulated tocotrienol has a low oral bioavailability. Self-emulsifying drug delivery systems (SEDDS) have been broadly used to promote the oral absorption of poorly water-soluble drugs, which may help to resolve the problem of low bioavailability for tocotrienol [3].This study aimed to evaluate the skeletal therapeutic effects of annatto tocotrienol treatment formulated with self-assembly drug delivery system in a rodent model of osteoporosis induced by oestrogen deficiency.

**Methodology**

Thirty six female Sprague Dawley rats aged 8 months were randomly assigned into six groups. The baseline group were euthanized upon receipt. All the groups except the sham underwent bilateral ovariectomy to induce oestrogen deficiency. The sham group was subjected to the same surgical stress, but their ovaries were retained. The rats were allowed to recuperate and to develop bone loss over the course of two months. After two months, treatment was initiated. The AnTT group received annatto tocotrienol at 60 mg/kg daily orally. The SEDDS-AnTT group received annatto tocotrienol prepared in SEDDS at 60 mg/kg daily orally. The positive control group received raloxifene 1 mg/kg daily orally. The remaining groups received equivolume of SEDDS without tocotrienol. After two months of treatment, the rats were euthanized and their left femurs were harvested. Structural changes of the femoral trabecular and cortical bone were examined using X-ray micro-computed tomography.

**Results and Discussion**

Oestrogen deficiency significantly caused deterioration of trabecular bone indices and cortical thickness in ovariectomized rats (p<0.05). Only AnTT formulated with SEDDS significantly prevented deterioration of trabecular bone (p<0.05). Both unformulated and formulated AnTT significantly improved cortical thickness in ovariectomized rats (p<0.05). No significant different were observed between formulated and unformulated AnTT in all parameters (p>0.05).

**Conclusion**

In conclusion, AnTT formulated with SEDDS is effective in reversing degeneration of the trabecular and cortical bone induced by oestrogen deficiency. Therefore, it is a potential antiosteoporotic agent for postmenopausal women.

**References**

[1] Riggs BL, et al. Sex steroids and the construction and conservation of the adult skeleton. Endocr. Rev. 2002, 23:279-302.

[2] Chin KY, et al. The biological effects of tocotrienol on bone: A review on evidence from rodent models. Drug. Des. Devel. Ther. 2015, 9:2049-2061.

[3] Alqahtani S, et al. Enhanced solubility and oral bioavailability of gamma-tocotrienol using a self-emulsifying drug delivery system (sedds). Lipids. 2014, 49: 819-829.

### OP31 Cross-talk of The Genes In HPV *E7* Knockdown HeLa and CaSki Cells

#### Muniandy,Kalaivani^1^, Mohana Kumaran,Nethia^2^, Shamsuddin,Shaharum^3^, Balakrishnan,Venugopal^1^

##### ^1^Institute for Research in Molecular Medicine (INFORMM), Universiti Sains Malaysia, Penang, Malaysia; ^2^School of Biological Sciences, Universiti Sains Malaysia, Universiti Sains Malaysia, Penang, Malaysia; ^3^School of Health Sciences, Universiti Sains Malaysia, Health Campus, Kubang Kerian, Kelantan, Malaysia

###### **Correspondence:** Balakrishnan,Venugopal (venugopal@usm.my)

**Background**

Alteration of the cellular signalling pathway affects cervical cancer progression. HPV manipulates the cellular signalling pathway for their transformation and the high-risk HPV *E7* oncogene have additional cancer associated activities by regulating signalling pathways. Alike other signalling pathways, the janus kinase-signal transducer and activator of transcription (*JAK/STAT*) pathway is an important regulator in HPV infected cervical cancer. However, not much work was published utilising HPV18 *E7* knockdown (KD) HeLa and HPV16 *E7* knockdown (KD) CaSki cells concerning the *JAK/STAT* signalling pathway. Therefore, this study was aimed to evaluate the regulation of genes in the *JAK/STAT* pathway in E7 knockdown HeLa and CaSki cells.

**Methodology**

HPV18 *E7* KD HeLa, pcDNA HeLa, HPV16 *E7* KD CaSki, pcDNA CaSki and pcDNA C33-A total RNA were subjected to nCounter pancancer pathway analysis.

**Results and Discussion**

*STAT 3* is always detected in elevated expression in the cervical cancer malignancy. Previous study demonstrated that CaSki exhibited high level of *STAT 3*, compared to HeLa and C33-A cell lines. In the *E7*-suppressed HeLa cells, the *STAT* expression was significantly down-regulated. Subsequently the suppressor of cytokine signalling (*SOCS*) and the *Bcl-2* anti-apoptotic gene were significantly up-regulated. The pro-apoptotic *Bcl-xl* expression exhibited down-regulation. *c-myc* gene expression was significantly down-regulated while *Cyclin D* demonstrated the similar expression as the pcDNA HeLa cells. Down-regulation of the, *p21* cyclin-dependent kinase inhibitor-1 was also observed in the suppression of HPV18 *E7* in HeLa cells. On the other hand, *JAK*, *STAT* and the *STAT* dimers demonstrated similar expression in the HPV16 *E7* suppressed CaSki cells. *SOCS, Bcl-2, Cyclin D* and *p21* showed unchanged expression. The *c-myc* gene and *Bcl-xl* gene were significantly down-regulated. The mechanism by which *E7* regulates *STAT 3* is unknown, however our data demonstrated, *STAT 3* was significantly down-regulated in the *E7* KD HeLa cells. When HPV18 *E7* KD HeLa cells expression data were normalised against C33-A cells, the data exhibited up-regulation of the phosphorylated *JAK*, however the cytoplasmic *STAT* and *STAT* dimers expressed similarly as the C33-A cells. Moreover, up-regulation of the *SOCS* were obtained which acts as the suppressor of the cytokine signalling. The expression of *p21, CDK1* and *Bcl-2* were significantly up-regulated but down-regulation of *Cyclin D* was observed. Both, *Bcl-xl* and *c-myc* showed similar expression as the C33-A cells. This clearly indicates that HPV18 *E7* regulates the *STAT* expression in HeLa cells, however further experiments are required to validate the interaction. In the *E7* suppressed CaSki cells, other oncogenes were found to regulate the expression of *STAT*. When HPV16 *E7* KD CaSki cells were normalised to the C33-A, the data demonstrated up-regulation of the *JNK*, *STAT* and *STAT* dimers. The expression of the *p21*, *Cyclin D, Bcl-xl* genes were significantly up-regulated while *c-myc* was down-regulated.

**Conclusion**

The information from this study will lead towards identification of molecules which play a role in cancer progression upon the suppression of *E7*. As multiple genes were activated by HPV, it is crucial to inhibit multiple molecules as a therapeutic option.

**References**

[1] Harrison, D. A. (2012). The JAK / STAT Pathway. *Cold Spring Harbor Perspectives in Biology, 4*(3), 1–4.

[2] Liang, Z., Gao, L. H., Cao, L. J., Feng, D. Y., Cao, Y., Luo, Q. Z., Li, M. (2012). Detection of STAT2 in early stage of cervical premalignancy and in cervical cancer. *Asian Pacific Journal of Tropical Medicine, 5*(9), 738–742.

[3] Shukla, S., Shishodia, G., Mahata, S., Hedau, S., Pandey, A., Bhambhani, S., Bharti, A. C. (2010). Aberrant expression and constitutive activation of STAT3 in cervical carcinogenesis: Implications in high-risk human papillomavirus infection. *Molecular Cancer, 9*(1), 282.

### OP32 Eponemycin Inhibits Ubiquitin Proteasome System of *Plasmodium falciparum* by Beta Subunit of 20S CP (in silico study)

#### Kana Mardhiyyah^1,2^, Sri Winarsih^3^, Loeki Enggar Fitri^4^, and Tatit Nurseta^5^

##### ^1^Doctoral Program in Medical Sciences, Faculty of Medicine Universitas Brawijaya, Malang, Indonesia; ^2^Department of Biochemistry and Biomolecular, Faculty of Medicine Universitas Brawijaya, Malang, Indonesia; ^3^Department of Pharmacy, Faculty of Medicine Universitas Brawijaya, Malang, Indonesia; ^4^Department of Parasitology, Faculty of Medicine Universitas Brawijaya, Malang, Indonesia; ^5^Departemnt of Obstetric and Gynecology, Faculty of Medicine Universitas Brawijaya, Malang, Indonesia.

###### **Correspondence:** Kana Mardhiyyah (kanamardhiyyah@ub.ac.id)

**Background**

Malaria is a tropical disease that still posed a problem in the world. Many antimalarial drug are already resistant to *Plasmodium*. Discovery of malaria drug candidate is becoming important recently. Proteasome in *Plasmodium* is now become the target in malaria drug candidate research. The aim of this study was to know the potency of eponemycin as Ubiquitin Proteasome System (UPS) inhibitor in beta subunit of 20S core particle (CP).

**Methodology**

The methods of this study were Protein Modelling (SWISS) and Molecular Docking (AUTODOCK VINA PYRX). Molecular docking was done specifically by simulating the binding site of selective drug that already bind (7f1).

**Results and Discussion**

The result showed there was affinity binding between eponemycin with beta subunit of 20S CP of *Plasmodium* UPS with scored -6.2, while 7F1 score was -6.7. The more negative binding affinity score, the easier ligand and receptor to bind. Score of eponemycin with beta subunit of 20S CP of *Plasmodium* UPS was approaching the selective drug, moreover this score was better than epoxomycin which is also a proteasome inhibitor.

**Conclusion**

The conclusion is eponemycin may become a candidate of antimalarial drug by its potency as UPS inhibitor in beta subunit of 20S CP of *Plasmodium* UPS.

### OP33 IL-6 and IL-10 levels in female patients with anti-Ro/La autoantibodies: A multicentred analysis in northern region of Malaysia

#### Anisah, Abdul Zubir^1,2^; Balqissiah, Baharudin^2^; Noor Shuhaila, Husain^3^; S Zulkafli, Nor Effa^1^

##### ^1^Regenerative Medicine Cluster, Advanced Medical and Dental Institute, Universiti Sains Malaysia, Bertam, 13200 Kepala Batas, Penang, Malaysia; ^2^Microbiology Unit, Pathology Department, Hospital Tuanku Fauziah, 01000 Kangar, Perlis, Malaysia; ^3^Inventory and Component Unit, North Regional Blood Centre, Hospital Sultanah Bahiyah II, 05100 Alor Setar, Kedah, Malaysia

###### **Correspondence:** Nor Effa (effa@usm.my)

**Background**

Autoantibodies formation has been greatly defined in the pathogenesis of autoimmunity. It has been used as diagnostic tools in clinical settings worldwide. Identification of corresponding cytokine mediators during autoantibody detection may herald putative therapeutic role in disease management. The aim of this study is to analyze IL-6 and IL-10 levels in female patients with anti-Ro/La autoantibodies from multicentered hospitals in the northern region of Malaysia.

**Methodology**

Sera samples were collected from Malaysian hospitals in the northern region and tested positive for connective tissue diseases (CTD). Healthy controls were sera collected from the Blood Bank of northern region. Cytokine levels were measured using Enzyme Linked Immunosorbent Assay (ELISA) with wavelength of 450 nm.

**Results and Discussion**

A significant increase in IL-6 levels were observed in patients with single positive anti-Ro and double positive anti-Ro/La autoantibodies compared to control group (70.56±115.59 pg/ml vs 20.48±15.19)(p<0.05). Although significant, IL-10 levels increased moderately in similar respondents as compared to control group (32.96±28.36 vs 17.10±12.08). Both cytokine levels subsided during disease progression in patients with single anti-Ro autoantibodies but not in double positive anti-Ro/La patients. Current data added scientific evidence to literature on correlation between CTD and IL-6 and IL-10 levels. In SLE patients, IL-6 [1] and IL-10 [2] levels spiked tremendously as compare to healthy control. This study demonstrated significant role of IL-6 and IL-10 in anti-Ro and anti-La autoantibodies formation among Malaysian population.

**Conclusion**

Association between these cytokines during the formation of anti-Ro and anti-Ro/La autoantibodies may indicate the underlying pro- and anti-inflammatory feedback responses. The high levels of IL-6 in single positive anti-Ro autoantibodies may be implicated in various type of CTD. Data from current study may pave way on manipulation of IL-6 inhibitors or IL-10 stimulants as therapeutic targets in CTD management among Malaysian population.

**References**

[1] Ripley BJ et al. Raised levels of interleukin 6 in systemic lupus erythematosus correlate with anaemia. Ann Rheum Dis. 2005;64(6):849–853. doi:10.1136/ard.2004.022681

[2] Abd Elazeem et al. Correlation of serum interleukin-10 level with disease activity and severity in systemic lupus erythematosus. Egyptian Rheumatology and Rehabilitation. 2018;45(1):25-33.

### OP34 Adjuvant-dependent peptide vaccination augments cellular immunity against latency tuberculosis antigen

#### Min Han Lew, Gee Jun Tye

##### Institute for Research in Molecular Medicine (INFORMM), Universiti Sains Malaysia, 11800 Minden, Penang, Malaysia

###### **Correspondence:** Gee Jun Tye (geejun@usm.my)

**Background**

Tuberculosis (TB) is a contagious lung disease caused by the infection of *Mycobacterium tuberculosis*, displaying a high mortality rate among human population. BCG vaccination is the only preventive measure to confer protection upon tuberculosis but only protects children with immunity waning over time. In this study, MHC-restricted peptides were designed to stimulate T-cell immunity against a latency-associated TB antigen, known as alpha-crystalline heat-shock protein (HspX). To further improve its immunogenicity, peptide vaccine was formulated in combined adjuvant for synergistic stimulation of cellular immunity (CASAC) in efforts to potentiate CD8+ T-cell mediated response. Previous study showed that CASAC adjuvant triggered activation of dendritic cells with stronger cell-mediated immunity [1]. Our study evaluated the adjuvanticity of CASAC on the enhancement of CD8+ T-cell population against HspX-specific MHC-restricted peptides.

**Methodology**

MHC-restricted peptides were predicted by Immune Epitope Database (IEDB) using α-crystalline HspX tuberculosis antigen (*Mycobacterium tuberculosis* Rv2031c strain, NP_216547.1) as template sequence. MHC-I and -II peptide with lower percentile rank (%) represented a higher tendency of MHC-binding to H-2-Kb and H-2-IAb allele, respectively. C57BL/6 mice (n = 5) were initially primed with recombinant HspX protein by intraperitoneal injection. After 14 days, the animals were intradermally immunised using MHC-I and -II peptides formulated in CASAC adjuvant, in every 2 weeks for 10 consecutive rounds. After resting for 50 days, mice were challenged to evaluate its recall immunity. Peripheral blood mononuclear cells (PBMC) were isolated for ELISA and Flow Cytometry analysis. Experimental data were plotted as mean ± standard error mean (SEM) using one-way ANOVA and unpaired *t*-test (two-tailed), where value *p* ≤ 0.05. Flow cytometry data were processed and analysed using FlowJo^TM^ v10 and GraphPad Prism 6 software.

**Results and Discussion**

CD8+ T-cell immunity is important when it comes to rationale TB vaccine design, owing to its function to stimulate cytotoxic T-lymphocyte reaction (CTL) that kills *M. tuberculosis* residing on infected tissues [2]*.* Our results demonstrated that CASAC adjuvant induced higher level of antigen-specific CD8+ T-cell response compare to mice group immunised with HspX alone. Effector memory (CD8^+^CD44^+^CD62L^-^) of CD8^+^ T-cell was improved with the use of CASAC adjuvant, while central memory (CD8^+^CD44^+^CD62L^+^) of all groups were sustained throughout peptide vaccination. The use of MHC-peptides in combination with CASAC has improved Th1-mediated response with elevated IFN-γ and TNF-α secretion with low IL-10 production. Immunosuppression was not apparent, as indicated by sustained level of regulatory T-cell (CD4^+^CD25^+^FoxP3^+^) population observed between group immunised with or without CASAC help. Only small percentage of KLRG1^+^ and PD1^+^ CD8+ T-lymphocyte population was stimulated with the use of CASAC formulation, indicating that CD8+ response was efficient without causing exhaustion and senescence of HspX-specific T-cells. Taken together, CASAC adjuvant ameliorated functional CD8+ T-cell response against HspX antigen, with least involvement of cellular negative feedback due to overstimulation of MHC-peptides.

**Conclusion**

The adjuvant-dependent MHC-restricted peptide vaccination generated efficient T-cell responses with least hindrance from immunosuppression, which might lead to an alternative therapeutic approach for TB.

**References**

[1] Wells, J.W., Cowled, C.J., Farzaneh, F., Noble, A., 2008. Combined triggering of dendritic cell receptors results in synergistic activation and potent cytotoxic immunity. Journal of immunology (Baltimore, Md.: 1950) 181, 3422-3431.

[2] Lin, P.L., Flynn, J.L., 2015. CD8 T-cells and *Mycobacterium tuberculosis* infection. Seminars in immunopathology 37, 239-249.

### OP35 Optimization of phage display technology for the selection and identification of dengue NS1 specific VNAR

#### Priyaanjali Loganathan^1^, Chiuan Yee Leow^2^, Zurina Hassan^3^, Chiuan Herng Leow^1,*^

##### ^1^Institute for Research in Molecular Medicine (INFORMM), Universiti Sains Malaysia, Penang, Malaysia; ^2^Institute for Research in Molecular Medicine (INFORMM), Universiti Sains Malaysia, Kubang Kerian, Kelantan, Malaysia; ^3^Centre for Drug Research (CDR), Universiti Sains Malaysia, Penang, Malaysia.

###### **Correspondence:**Chiuan Herng Leow(herng.leow@usm.my)

**Background**

Dengue, including dengue fever (DF), dengue haemorrhagic fever (DHF) and dengue shock syndrome (DSS), is among the major causes of morbidity and mortality. Dengue is an acute viral illness caused by RNA virus of the family Flaviviridae and spread by Aedes mosquitoes. The recent emergence of other arboviruses, such as Zika and Chikungunya has brought new challenges mainly due to the similarities among these diseases. It can hamper the correct diagnosis and management of patients. The conventional method of using IgM and IgG antibodies in patient serum delays the diagnosis of dengue as these antibodies are not reliable during the earlier stage of infection. Delay in the diagnosing of dengue in patients is the major cause of morbidity and mortality. As an alternative, NS1 antigen which is detectable in blood from the first day up to day 9 to 18. However, the onset of symptoms can be used to diagnose dengue in patients during the earlier stage of infection. Antibodies are exploited as binders for antigen of interest in a range of platforms ^1^. Monoclonal antibodies (mAbs) have been widely used for therapeutic, diagnostic, and biotechnological applications. Recently, the single-domain immunoglobulins such as the shark VNAR (new antigen receptor variable domain) have exhibited to possess excellent solubility and high thermostability. This research focuses on screening phage display libraries to identify the clone recognizing to dengue NS1 antigen. Binders specific to proteins of interest with high affinity can be selected by biopanning. The clone targeting the NS1 antigen is expected to be isolated. The isolated NS1 antigen can be used to diagnose dengue in patients during earlier stages of infection and is expected to reduce the rate of morbidity and mortality.

**Methodology**

Using semi-synthethic phage library from shark, biopanning was done to isolate antibodies against NS1 antigen. Briefly, 100 μg, 50 μg, and 25 μg of NS1 antigen was coated on three immunotubes. After blocking with 2% MPBS, 1 ml of VNAR phage library (1.1 × 10^13^ pfu/ml) was inoculated to first immunotube for biopanning process. The eluted phage was then rescued with appropriate amount of helper phage prior to amplification for next rounds of panning. The panned antibodies were verified by polyclonal phage ELISA.

**Results and Discussion**

Optimization for the growth of TG1 *E.coli* was verified from two media, namely 2YT and M9TB. After completing 3 rounds of biopanning, the amplified phage obtained from end of each round of biopanning was tested for the presence of antibody against NS1 antigen using polyclonal phage ELISA. It was observed that there was enrichment of antibody in round 2 and round 3 of biopanning with 2-fold higher when compared to that of than pre-library.

**Conclusion**

Enrichment of antibody against NS1 antigen was successfully done. The experiment will further be continued with monoclonal phage ELISA. The monoclonal clone specific to recombinant DENV Type 2 NS1 will then be identified through DNA sequencing.

**References**

[1] Leow, C. H. *et al.* Isolation and characterization of malaria PfHRP2 specific VNAR antibody fragments from immunized shark phage display library. *Malaria Journal* **17**, 383 (2018).

### OP36 The Inflammatory Biomarker of Interleukin-6 and Metabolic Syndrome in Rats with High Fat Diet

#### Zulhabri Othman^1^, Jamunarani Murugan^1^, Maleeka Abdullah Hilmy^1^ and Muhammad. Fakhruddin Irfan Sazali^1^

##### ^1^School of Graduate Studies, Post-Graduate Centre, Management and Science University, MALAYSIA

*BMC Proceedings* 2019, **Vol (Suppl no.):**P1

###### **Correspondence:** Zulhabri Othman (zulhabri_othman@msu.edu.my)

**Background**

The metabolic syndrome (MetS) is a cluster of parts reflecting oversupply, sedentary lifestyles, and resulting surplus adiposity. The MetS involves the clustering of abdominal obesity, insulin resistance, dyslipidemia, and increased blood pressure and is linked with other co morbidities including pro thrombotic state, pro inflammatory state, non-alcoholic fatty liver illness, and reproductive disorders [1]. Interleukin 6 (IL-6), generated quickly and transiently in reaction to infection and tissue injury, adds to host defense by stimulating acute phase reactions, hematopoietic, and immune reactions. In the initial stage of inflammation, after IL-6 is produced in a local lesion, it passes through the bloodstream to the liver [2]. This study aims to investigate the association of IL-6 and MetS characteristics in rats with high-fat diet.

**Methodology**

Eight-week-old Sprague-Dawley Rat (n=15) male rats were divided into three groups, high-fat diet (HFD), normal diet (ND) and restrictive diet (RD). The rats were kept in a controlled environment (22–24 ° C and 12 h light / dark cycles) and given with specific diets for each group for 12 weeks [3]. The body weight, glucose and total cholesterol level were monitored. Blood was collected (2mL) for glucose and total cholesterol analysis. The inflammatory biomarker IL-6 was analysed using ELISA kit.

**Results and Discussion**

The body weight, glucose level and total cholesterol of the rats were increasing every 30 days. Results have shown a significant different (p<0.0001; F=45.95) of body weight between groups, with HFD (451.74 ±45.56 g) recorded the most weighted rats compared to ND (271.06 ±10.50 g) and RD (263.14 ±6.53 g) groups. The glucose level was shown a significant difference (p<0.0001; F=67.84) between groups, with mean glucose for HFD increased to 132.33 ±10.96 mg/ml follows with ND (94.67 ±1.15 mg/ml) and RD (72.33 ± 0.58 mg/ml) groups. The total cholesterol also shown a significant difference (p<0.0001; F=2623.0) between groups. Mean total cholesterol for HFD was 240.66 ± 5.03 mg/ml compared to ND (96.00 ± 0.01 mg/ml) and RD (88.00 ± 0.01 mg/ml) groups. The interlukin-6 was found to be higher in HFD (976.25 ±73.158 pg/ml) compared to ND (761.25 ±121.097 pg/ml) and RD (721.25 ±128.509 pg/ml) groups, but with no significant difference (p=0.004; F=10.08).

**Conclusion**

Consumption of high fat diet exhibit MetS characteristics such as increase in body weight, glucose level and total cholesterol. The elevated level of inflammation biomarkers IL-6 might be a potential biomarker for early prediction of any changes in metabolic syndrome or disorders that could cause by high-fat diet.

**Acknowledgement**

This study was funded by MOE FRGS grant (FRGS/1/2018/SKK08/02/2).

**References**

[1] Marc-Andre Cornier, et al., The Metabolic Syndrome, *Endocrine Reviews*, Volume 29, Issue 7, 1 December 2008, Pages 777–822, 10.1210/er.2008-0024

[2] Tanaka, T., et al., (2019). IL-6 in Inflammation, Immunity, and Disease. Retrieved 7 September 2019, from https://www.ncbi.nlm.nih.gov/pmc/articles/PMC4176007/

[3] Udomkasemsab, A., et al., (2018). High fat diet for induced dyslipidemia and cardiac pathological alterations in Wistar rats compared to Sprague Dawley rats. *Clínica E Investigación En Arteriosclerosis*, *31*(2), 56-62. doi: 10.1016/j.arteri.2018.09.004

### PP01 The effects of Goniothalamin on the expression of apoptosis and proliferative genes related to oral cancer using oral cancer animal model

#### Rola Ali-Saeed^1^, Aied M Alabsi^1^, Fahmi Kaid^2^

##### ^1^Faculty of Dentistry, MAHSA University, Bandar Saujana Putra, 42610 Jenjarom Kuala Langat, Selangor. Malaysia; ^2^Department of Oral and Craniofacial Sciences, Faculty of Dentistry, University of Malaya, 50603 Kuala Lumpur, Malaysia.

###### **Correspondence:** Rola Ali-Saeed (rolaali@mahsa.edu.my, rola_absi@yahoo.com)

**Background**

Oral squamous cell carcinoma (OSCC) is the sixth most common malignancy worldwide and the 5-year survival rate of around 50% has not improved significantly during the past 30 years. Chemotherapy in combination with anticancer agents derived from natural products offers a promising new approach in an attempt to improve patient prognosis [1]. Goniothalamin is a styryl-lactone compound, which was extracted from *Goniothalamus macrophyllus*. It was reported to be cytotoxic towards several tumors [2]. In this study, the evaluation of the Goniothalamin chemotherapeutic activity on OSCC cells *in vivo* was carried out using 4NQO-animal model.

**Methodology**

In this study, thirty-five SD rats were divided randomly into five groups of seven rats per each. Four groups were supplied with 4NQO (20 ppm) in their drinking water for 8 weeks [3]. Goniothalamin was administered orally to the rats a week before exposure to the carcinogen (4NQO) until a week after termination of the carcinogen exposure. All rats were sacrificed, and histological analysis was performed to assess any incidence of pathological changes. The expression of selected genes involved in apoptosis and proliferative mechanism related to oral cancer were evaluated using RT2-PCR.

**Results and Discussion**

The results obtained showed that the incidence of OSCC induced by 4NQO in rats receiving Goniothalamin was decreased, where smaller tumours were developed, compared to the untreated group. Goniothalamin induced apoptosis by upregulation of Casp3 and Bax genes and downregulation of Bcl-2, Cyclin D1, Tp53, EGFR and Cox-2 genes when compared to the induced cancer group.

**Conclusion**

The results of this study indicate that Goniothalamin has the promise to be developed as novel therapeutic agents for the treatment of OSCC.

**References**

[1] Jiao C, Xie YZ, Yang X, Li H, Li XM, Pan HH, Cai MH, Zhong HM, Yang BB. (2013). Anticancer activity of Amauroderma rude. PloS One, 8(6), e66504.

[2] Alabsi AM, Ali R, Ali AM, Al-Dubai SA, Harun H, Abu Kasim NH, Alsalahi A. (2012). Apoptosis induction, cell cycle arrest and in vitro anticancer activity of gonothalamin in a cancer cell lines. Asian Pacific journal of cancer prevention. 13: 5131-5136.

[3] McCormick DL, Horn TL, Johnson WD, Peng X, Lubet RA, Steele VE. (2015). Suppression of Rat Oral Carcinogenesis by Agonists of Peroxisome Proliferator Activated Receptor γ. PloS one, 10(10), e0141849.

### PP02 Potential of recombinant LipL21 as ELISA antigen for screening of human leptospirosis

#### Mohammad Ridhuan Mohd Ali^1^, Nur Mukmina Dasiman^2^ and Fairuz Amran^2^

##### ^1^Bacteriology Unit, Infectious Disease Research Centre (IDRC), Institute for Medical Research (IMR), Ministry of Health Malaysia, National Institutes of Health Complex, Bandar Setia Alam, 40170 Shah Alam, Selangor, Malaysia; ^2^Bacteriology Unit, Infectious Disease Research Centre (IDRC), Institute for Medical Research (IMR), Ministry of Health Malaysia, Jalan Pahang, 50588 Kuala Lumpur.

###### **Correspondence:** Fairuz Amran (fairuz@imr.gov.my)

**Background**

Accurate diagnosis of leptospirosis is important for patient’s prognosis. To date, many of the reported serological assays portray inconsistent sensitivity and specificity, depending on the study sites. Due to this limitation, a diagnostic test that can detect locally circulating *Leptospira* serovars in highly anticipated.

**Methodology**

Partial LipL21 gene from *Leptospira interrogans* serovar Lai was cloned and expressed in *E. coli*. Following purification, the rLipL21 was coated on the microtiter plates and used as ELISA antigen. Diluted goat, anti-human IgG/IgM antibodies conjugated with HRP was used as the secondary antibody. The cut-off point was set at 0.200 (OD_450 nm_). Eleven MAT-positive sera and eleven MAT-negative sera were tested in duplicates.

**Results and Discussion**

Twenty-two sera from leptospirosis suspected patients were tested. Based on the MAT status, the sensitivity and specificity of the developed ELISA was 90.9% and 90%, respectively. This finding showed that infected patients produced a detectable amount of anti-LipL21 IgG/IgM antibodies. Moreover, based on this serological result, it is assumed that the expressed recombinant protein fold correctly, similar to its native counterpart [1]. The minor discrepancies between MAT and the developed ELISA may be attributed to the low titer of antibodies or low level of LipL21 expression of the infecting *Leptospira* strains.

**Conclusion**

Based on this preliminary data, the developed ELISA detecting anti-rLipL21 human antibodies is sensitive and specific for the diagnosis of human leptospirosis. Further evaluation using a larger number of clinical specimens is needed in order to confirm the performance.

**References**

[1] Éverton F. Silva, et al. The terminal portion of leptospiral immunoglobulin-like protein LigA confers protective immunity against lethal infection in the hamster model of leptospirosis. Vaccine. Vol. 25 2007. Issue 33. Pg. 6277-6286.

[2] Monica Vieira, et al. *Leptospira interrogans* outer membrane protein LipL21 is a potent inhibitor of neutrophil myeloperoxidase. Virulence. Vol. 9 2018. Issue 1. Pg. 414-425

### PP03 Stem extract of *Strobilanthus crispus* inhibits breast cancer cells by inducing apoptosis

#### Norasyidah Gordani^1^ and Teoh Peik Lin^1^

##### ^1^Biotechnology Research Institute, Universiti Malaysia Sabah, Kota Kinabalu, Sabah, Malaysia

###### **Correspondence:** Teoh Peik Lin (peiklin@ums.edu.my)

**Background**

*Strobilanthes crispus* (*S. crispus*) is known to possess multiple health beneficial effects and has been widely used as traditional medicine for various ailments, such as cancer, gastrointestinal disorder, and wound healing. In Malaysia, it is called “pecah kaca” or “jin batu”. This study was to investigate whether its stem extract also exhibiting antiproliferative activity on breast cancer cells.

**Methodology**

The plant was grown by the herbal supplier in Kota Kinabalu and verified by a botanist from Universiti Malaysia Sabah (ACSC 001/2013). The leaves and stem were washed and dried before freeze-drying. The chemical compounds were first extracted using methanol for 13 hours followed by liquid-liquid partition using hexane, chloroform, ethyl acetate, and water solvents. The antiproliferative activity of these extracts was tested on breast cancer cell line, MDA-MB-231 using MTT assay. Morphological changes caused by the treatment was studied using methylene blue and DAPI staining. Then, the mode of action of the potential extracts was examined through gene and protein expression of genes associated with apoptosis and cell cycle.

**Results and Discussion**

The MTT results showed two extracts of *S. crispus* inhibited the proliferation of MDA-MB-231 at the IC_50_ of 40 μg/ml and 60 μg/ml after treated with leaf water (LW) and stem hexane (SH) extracts, respectively. The discrepancy of IC_50_ values compared to other studies could be due to the differences in extraction methods [1]. Chromatin condensation and peripheral aggregation of nuclear chromatin were also observed in treated cells. The SH extract was found to induce apoptosis in MDA-MB-231 by suppressing BCL-2 and promoting expression of apoptotic genes. Moreover, a decrease of cyclin A2 expression suggested potential dysregulation of the cell cycle. In contrast, LW extract showed no clear occurrence of apoptosis and cell cycle arrest. This is not surprising as different extracts may contain a different composition of bioactive compound(s). Anticancer and apoptotic inducing properties of leaf extracts of *S. crispus* have also been reported in many studies [2][3].

**Conclusion**

Taken together, these results suggested that partitioned LW and SH extracts of *S. crispus* might prevent cancer cell growth through different mechanisms.

**References**

[1] Bimakr M., et al. Comparison of different extraction methods for the extraction of major bioactive flavonoid compounds from spearmint (*Mentha spicata* L.) leaves. Food and Bioproducts Processing. 2011; 89(1), 67-72.

[2] Asmah R., et al. Anticarcinogenic properties of *Strobilanthes crispus* extracts and its compounds *in vitro*. International Journal of Cancer Research. 2006; 2(1), 47-49.

[3] Yaacob N.S., et al. Anticancer activity of a sub-fraction of dichloromethane extract of *Strobilanthes crispus* on human breast and prostate cancer cells *in vitro*. BMC Complement Altern Med. 2010; 10, 42.

### PP04 Mutational analysis of *CALR* using conformational sensitive gel electrophoresis (CSGE) in *BCR-ABL* negative myeloproliferative neoplasm patients

#### Nur Atikah Zakaria^1,2^, Norfifiana Alisa Rosle^1,3^, Azie Sudin^1^, Haiyuni Mohd Yassim^1^, Nurul Ameera Samat^1^, Ibrahim Khidir Ibrahim^1,4^, Rosline Hassan^1^, Marini Ramli^1^, Shafini Mohamed Yusoff^1^, Azlan Husin^5^ and Muhammad Farid Johan^1^

##### ^1^Department of Haematology, School of Medical Sciences, Universiti Sains Malaysia, Kelantan, Malaysia; ^2^School of Health Sciences, Universiti Sains Malaysia, Kelantan, Malaysia; ^3^Faculty of Applied Science, Universiti Teknologi MARA, Selangor, Malaysia; ^4^Department of Haematology, Faculty of Medical Laboratory Sciences, Al-Neelain University, Khartoum, Sudan; ^5^Department of Medicine, School of Medical Sciences, Universiti Sains Malaysia, Kelantan, Malaysia.

###### **Correspondence:** Muhammad Farid Johan (faridjohan@usm.my)

**Background**

Mutations in calreticulin (*CALR*) have been reported to be key markers in the molecular diagnosis of myeloproliferative neoplasms. Mutations in *CALR* are commonly found in essential thrombocythemia (ET) and primary myelofibrosis (PMF) patients lacking *JAK2* mutations. The presence of *CALR* mutations is a critical diagnostic criteria for myeloproliferative neoplasm hence, the detection of *CALR* mutations increases the diagnostic accuracy in patients without other molecular markers. In most previous reports, *CALR* mutations were analyzed by allele-specific PCR (AS-PCR) and Sanger sequencing. Here, we report conformational sensitive gel electrophoresis (CSGE) as a screening tool for *CALR* mutation in *BCR-ABL* negative myeloproliferative neoplasms (MPN) patients.

**Methodology**

PCR primers were designed to amplify 537 bp spanning between exons 8 and 9 to target the mutation hot spot in *CALR*. Twenty nanograms of DNA template and 0.5 μL (10 pmol) of each primer were added to the PCR premix (25 μL final volume) prior amplified by PCR. Gel electrophoresis was performed in a 2% agarose gel to detect the amplified products. CSGE was used to screen for mutations as described previously [1]. Briefly, PCR products were denatured by heating to 95°C for 5 min and then incubated at 65°C for 30 min. These heteroduplexed PCR products were then electrophoresed on 10% polyacrylamide gels; 40% 29:1 acrylamide/bis (Bio-Rad), 10% ethylene glycol (Sigma-Aldrich), 15% formamide (Sigma-Aldrich) and 0.5 x TTE buffer (1 x TTE = 89 mmol/L Tris, 28.5 mmol/L taurine, 0.2 mmol/L EDTA). Samples displaying an abnormal CSGE profile, when compared with that obtained from a normal individual, were directly sequenced. The results were confirmed by Sanger sequencing and compared with results from AS-PCR as described previously [2].

**Results and Discussion**

Forty peripheral blood samples from *BCR-ABL* negative MPN patients with a mean age of 56.7±11.5 years were analyzed for *CALR* using CSGE and AS-PCR. They include 19 PV, 7 ET, and 14 PMF. CSGE identified three types of mutations; two PMF patients with either *CALR* type 1 or type 2, and additional one PV patient with p.K368del (c.1102_1104delAAG) in frame mutation albeit previous publications showed none in this group. All three mutated patients have altered KDEL motif at the C-terminal of CALR protein. However, CSGE did not detect one ET patient with type 1 mutation as detected in AS-PCR.

**Conclusion**

Although CSGE did not detect one patient with mutation as detected in AS-PCR, CSGE detected an extra mutation and may be considered as more sensitive and accurate in detecting *CALR* mutation in *BCR-ABL* negative MPN patients.

**References**

[1] Williams IJ, et al. Conformation-sensitive gel electrophoresis. In: Theophilus BDM, et al. (eds). PCR Mutation Detection Protocols. New Jersey, Humana Press, 2002; p. 137-150.

[2] Jeong JH, et al. Screening PCR Versus Sanger Sequencing: Detection of CALR Mutations in Patients With Thrombocytosis. Ann. Lab. Med. 2016; 36:291-299.

### PP05 Identification of Functional Variable Regions of Antibody Transcripts derived from Mouse Hybridoma Cells for the Generation of Chimeric Antibodies

#### Romchat Kraivong^1^, Prasit Luangaram^1^, Narodom Phaenthaisong^1^, Prida Malasit^1,2^, Watchara Kasinrerk^3,4^, Chunya Puttikhunt^1,2^

##### ^1^Medical Biotechnology Research Unit, National Center for Genetic Engineering and Biotechnology (BIOTEC), National Science and Technology Development Agency (NSTDA), Bangkok, Thailand; ^2^Division of Dengue Hemorrhagic Fever Research, Department of Research and Development, Faculty of Medicine Siriraj Hospital, Mahidol University, Bangkok, Thailand; ^3^Biomedical Technology Research Center, National Center for Genetic Engineering and Biotechnology (BIOTEC), National Science and Technology Development Agency (NSTDA), Chiang Mai, Thailand; ^4^Division of Clinical Immunology, Department of Medical Technology, Faculty of Associated Medical Sciences, Chiang Mai University, Chiang Mai, Thailand.

###### **Correspondence:** Chunya Puttikhunt (chunyapk@biotec.or.th)

**Background**

Mouse monoclonal antibodies (mAbs) specific to target epitopes/proteins of interest are produced from immortal hybridoma cell lines. However, genes encoding functional variable kappa light chain (Vκ) of mAbs are proportionally much less than that of the aberrant ones in murine hybridoma cells derived from MOPC21 myeloma fusion partners. This causes a bias toward the amplification of functional Vκ gene and hampers subsequent antibody engineering process. Here we report the efficient protocols to select and verify the functional Vκ genes as well as functional variable heavy chain (VH) transcripts from 17 hybridoma cells producing mAbs against dengue proteins.

**Methodology**

PCR amplification of the first strand cDNA using a set of different sense primers covering most of mouse immunoglobulin families, subsequently with *Bci*VI restriction enzyme digestion enabled identification of undigested 400-bp PCR products as productive Vκ transcripts based on sequencing analysis. In addition, the 500-bp VH transcripts were amplified by the other set of primers from all hybridomas. Productive Vκ and VH were generated in a form of chimeric antibodies as a proof-of-concept. DNA sequences encoding murine variable regions and human constant regions specific for IgG1 were assembled into a dual mammalian expression vector. The binding characteristics of the chimeric antibodies were tested by immunoblot and immunofluorescence assays.

**Results and Discussion**

The 400-bp PCR products of Vκ transcripts were amplified by Vκ primers from 17 hybridoma cells producing anti-dengue mAbs, and subsequently digested with *Bci*VI. Only the *Bci*VI-resistant products were identified as productive Vκ transcripts, confirming by sequencing analysis. The productive 500-bp VH transcripts were also amplified from all hybridomas, except the clone 1B2 where a frame shift at the CDR3 region was detected. Productive Vκ and VH transcripts of three hybridoma clones (2G6, 1F11 and 1A4) were selected for the generation of functional chimeric antibodies. The binding characteristics of these chimeric antibodies were similar to the original hybridoma antibodies.

**Conclusion**

In this study, the simple and efficient protocol to identify productive variable genes from mouse hybridoma clones was demonstrated and subsequently verified by the generation of functional chimeric antibodies. It is beneficial to generate chimeric antibodies for various applications.

**References**

[1] Carroll, W. L., Mendel, E., and Levy, S. Hybridoma fusion cell lines contain an aberrant kappa transcript. Molecular immunology. 1988, 25: 991-995.

[2] Ding, G., Chen, X., Zhu, J., and Cao, B. Identification of two aberrant transcripts derived from a hybridoma with amplification of functional immunoglobulin variable genes. Cellular & molecular immunology. 2010, 7: 349-354.

### PP06 Quantitative proteomics analysis of oil palm root during early stages of *Ganoderma* inoculation

#### Shahwan, S; Othman, A; Dzulkafli, S, B; Hassan, H; Idris A. S, I. A; Amiruddin, M. D and Ramli, U.S.

##### Malaysian Palm Oil Board, No. 6, Persiaran Institusi, Bandar Baru Bangi, 43000 Kajang, Selangor, Malaysia

###### **Correspondence:** Shahwan, S (syahanim@mpob.gov.my)

**Background**

Oil palm is prone to fungal attack by basal stem rot (BSR) disease, a serious threat to oil palm plantation. The basidiomycete fungus, *Ganoderma boninense*, degrading the root tissue beneath the soil and later became hemibiotroph, destroying the oil palm and affecting the productivity of the oil palm. Five to ten years later, the physical symptom of unopened spear leaf and presence of fruiting body can only be seen and eventually the palm will die. Biochemical studies via quantitative proteomics were engaged to catalogue differentially expressed proteins that was being triggered during early interaction of *Ganoderma* in oil palm.

**Methodology**

The 12-month old oil palm seedlings were artificially inoculated with *G. boninense* inoculum (T1) using root inoculation technique as described by Idris (1999) with modification [1]. Control experiments were employed i.e. (T2) oil palm seedlings inoculated with rubber wood block (RWB) without *G. boninense* and (T3) oil palm seedlings uninoculated. Roots were collected at day 3 and day 7 after inoculation and cleaned prior to snap frozen until further used. Phenol extraction method was conducted to extract total proteins from oil palm root [2]. Proteins were precipitated in 0.1 M Ammonium bicarbonate and resuspended in 0.1 M TEAB prior to quantification via 2D Quant Kit. Offline reverse phase fractionation was performed prior to LC-MS analysis via Orbitrap Fusion. The mass spectrometry spectrum was analyzed via Thermo Scientific^TM^ Proteome Discoverer^TM^ Software Version 2.1 using NCBI Oil Palm Protein and *Ganoderma* Protein databases. All peptides were validated using the percolator® algorithm, based on q-value less than 1% false discovery rate (FDR). Descriptive analysis was performed via Perseus Software Version 1.6.2.1**.** Each experiment was conducted in duplicate.

**Results and Discussion**

Physically, inoculated oil palm looks healthy. At day 3 post inoculation, no visible symptoms were seen at the oil palm root. Only after day 7 post inoculation, white mycelia of *Ganoderma* was observed surrounding the root skin. Six fractions of labelled oil palm root proteome at day 3 and day 7 were collected and generated a total of 248253 MS/MS spectrum. Peptide spectrum matching towards selected databases resulted to a total 8936 PSMs with 1207 protein groups identified. After filtration, a total of 502 proteins was valid for quantitative analysis. To explore the similarities and comparative proteome profiles in oil palm root in response to *Ganoderma* at early stages of inoculation, pairwise comparison was carried out between the proteomes responding to *Ganoderma* at day 3 and day 7 (labelled with tag 126 and 129, respectively) and empty wood block as control at day 3 and 7 (labelled with tag 127 and 130, respectively). A signal ratio of 126//127 and 129/130 was used to indicate the oil palm root proteins responsive to *Ganoderma* inoculation at day 3 and day 7, respectively. Using the given cutoffs, a total of 92 proteins were differentially expressed in the oil palm root inoculated with *Ganoderma* as early as day 3 after inoculation, with 59 up-regulated and 33 down-regulated proteins. Out of these, five proteins were significantly (p<0.05) regulated which include ricin-like proteins and xyloglucan galactosyl-transferase that interlace cellulase microfibrils in flowering plants [3]. At day 7, 36 proteins were differentially expressed in the oil palm root inoculated with *Ganoderma*, with 23 up-regulated and 13 down-regulated proteins. Out of these, two proteins were significantly regulated (p<0.05) i.e. chloroplastic small heat shock protein and pyrophosphate-fructose-6-phosphate 1-phosphotransferase subunit alpha.

**Conclusion**

This paper reported quantitative proteomics analysis employing oil palm root to reveal early interaction and differentially expressed proteins during *Ganoderma boninense* inoculation.

**References**

[1] Idris, A S. PhD thesis, Wye College, University of London, U. K. 1999.

[2] Syahanim et al*.* Journal of Oil Palm Research. 2013, 25:298-304.

[3] Madson et al. The Plant Cell. 2003, 15:1662-1670.

### PP07 Chemoresistant triple negative breast cancer (TNBC) subtype is mediated by miR-205 through activation of the TGFβ signaling pathway

#### Ezanee Azlina Mohamad Hanif^1^ and Amalia Afzan Saperi^1^

##### ^1^UKM Medical Molecular Biology Institute (UMBI),Universiti Kebangsaan Malaysia, Malaysia

###### **Correspondence:** Ezanee Azlina Mohamad Hanif (ezanee.azlina.mohamad.hanif@ppukm.ukm.edu.my)

**Background**

Relapse in triple negative breast cancer patients is common as a consequential by being chemoresistant to FEC chemotherapy. In clinical settings, this is still an underlying setback in TNBC treatment regime. Pinpoint signature markers and understanding molecular mechanisms in TNBC is crucial. This study aimed to pinpoint epithelial-to-mesenchymal transition (EMT) signatures and to establish the epigenetic molecular mechanism in TNBC cell lines.

**Methodology**

A total of 58 epigenetic enzymes were assayed via siRNA knockdown in Hs578T cell line to identify the epigenetic regulator playing an oncogenic key role in TNBC cell line. The least viability esiRNA was chosen for further evaluation. Because it is known that EMT is the main feature of TNBC progression, Nanostring Cancer Progression panel was carried out to determine differentially expressed genes in SETD1A depleted cells. Significant genes were further validated by RqPCR to determine the potential mechanism, with clonogenic and wound scratch assay to complement the regulation of genes in TNBC cell lines.

**Results and Discussion**

The epigenetic screening showed SETD1A as a potential regulator of cell proliferation and increased in TNBC cell line. Nanostring cancer panel highlighted several genes denoted as up- and down-regulated at a cut-off fold-change >1.5 and <-1.5 with p-value (p=<0.05) fall within key biological processes, narrowing down to top 20 upregulated (cell adhesion and cell cycle) and top 20 downregulated genes (EMT). The top 20 upregulated genes were COL5A1, CDH2, COL6A2, SULF1, COL4A1, SNAI2, FSTL1, VIM, VCAN, TXNIP, FHL1, CADM1, FBN1, JAM3, AKAP12, SMAD4, FBN2, EMP3, and ABI3BP. Top 20 differentially down-regulated genes were TACSTD2, S100A7, KRT19, SLP1, ELF3, AP1M2, ESRP1, TMPRSS2, ST14, SCNN1A, MGP, EPCAM, TSPAN1, B3GNT3, S100A14, TMEM30B, LRG1, CLDN4, TNFSF10, TMPRSS4 and SPDEF. Interestingly some of these genes are associated as active transcription factors that play a vital role in chemo-drug resistance in cancers. KEGG pathway analyses illustrated multiple pathways involved by the changes in gene expression profiles between the two cell lines. The distinct pathway involved by the regulated genes was the Pathway in Cancer, illustrated the big picture of several intrinsic pathways involved in cancer, such as the TGFβ pathway. The significant differential gene expression analyses were also associated with a pathway in miRNAs, depicting activation of gene changes (CD44, ITGA5, RhoA, uPA) regulated by several miRNAs indicating the roles of miRNAs (miR-373, miR-31, miR-193b, miR-200) as oncomiRNA in the breast cancer tumorigenesis. The clonogenic assay exhibited reduced proliferative and migration effects upon SETD1A-KD. RqPCR data showed significant increased of miR205 (p = 0.003) and LRG1 (p = 0.02) and reduction of Ki-67 (p = 0.02) expression level upon SETD1A-KD.

**Conclusion**

Our data suggests that SETD1A modulates proliferation through de-activation of miR-205 to regulate Ki-67, concurrently suppressed LRG1 through activation of the TGFβ signaling pathway in TNBC. Clearly SETD1A epigenetically abrogate EMT markers, thus, further assessments are warranted to elucidate the significance of SETD1A in TNBC chemo-resistance.

### PP08 *In vitro* anti-ovarian cancer and toxicity effects of water soluble standardized extract of *Eurycoma longifolia* (SEEL30)

#### Nurhanan Murni Yunos^1^, Muhammad Haffiz Jauri^2^, Chee Beng Jin^1^, Nor Datiakma Mat Amin^1^ and Nor Jannah Salehuddin^1^

##### ^1^Bioactivity Programme, Natural Products Division, Forest Research Institute Malaysia (FRIM), 52109, Kepong, Selangor, Malaysia; ^2^Phytochemistry Programme, Natural Products Division, FRIM, 52109, Kepong, Selangor, Malaysia.

###### **Correspondence:** Nurhanan Murni Yunos (hanan@frim.gov.my)

**Background**

Ovarian cancer (OC) still remains as one of the deadliest of gynaecological type of cancers. OC was often being detected at a late stage and this had reduced the chance of recovery. Chemotherapy such as cisplatin and paclitaxel are among the drugs that are still being used to treat the advanced stage of ovarian cancer but the prognosis is still poor due to drug resistance and adverse toxicity effects. Hence, the search for new anti-cancer agent is still in need to overcome these issues. *E. longifolia* had been reported previously to have anti-proliferative effects in epidermoid (KB), prostate (DU-145), rhabdomyosarcoma (RD), breast (MCF-7) cancer cell lines, to name a few [1]. However, the preparation of these extracts involved the usage of medium polar solvents (ethanol, methanol, and chloroform) and one of the active compounds detected was 9-methoxycanthin-6-one. Recently, a water soluble standardized extract (SEEL30) had been currently prepared using proprietary extraction protocol with a compound coded as EL30 was used as one of its biomarker.

**Methodology**

The roots of *Eurycoma longifolia* was harvested from Stesyen Penyelidikan FRIM in Maran, Pahang and it was extracted with distilled water using a proprietary extraction protocol (patent pending; UI2018703747) to yield a standardized water extract (SEEL30). HPLC was used to quantitate the percentage of active compound coded as EL30 in SEEL30. Different concentrations of SEEL30 (1, 10, 20, 50, 100 μg/mL) and EL30 (0.032, 0.16, 0.8, 4, 20 μg/mL) were treated on the SKOV-3 and A2780 ovarian cancer cells in 96-well plates and incubated for 72 hr in 5% carbon dioxide in air. Sulphorhodamine B assay was used to determine the IC_50_ values of SEEL30 [2]. Hoescht 33342 assay was used to determine the dose-dependent effect of SEEL30 in causing apoptotic cell death based on Apoptotic Indices (AI) analysis [3]. Bacterial Reverse Mutation Test (AMES) test (OECD471 Guidelines for Testing of Chemicals 1997) was used to evaluate the genotoxicity effect of different concentrations of SEEL30 (from 313 to 5000 μg/plate) on the bacterial strains *Salmonella typhimurium* (TA100, TA1535, TA98, TA1537) and *Escherichia coli* (WP2uvrA).

**Results and Discussion**

The yield of SEEL 30 was 4.49 ± 0.08 % from the dried roots of *E. longifolia*. 1.68 ± 0.17 % of EL30 was detected in SEEL30. The IC_50_ values of SEEL30 when treated in SKOV-3 and A2780 were 6.74 ± 0.04 μg/ mL and 8.93 ± 0.31μg/ mL, respectively. The IC_50_ values of EL30 when treated in SKOV-3 and A2780 were 1.26 ± 0.07 μg/ mL and 0.82 ± 0.07 μg/ mL, respectively. As for comparison, cisplatin was shown to be twice less active than EL30 when treated on the respective cell lines. It was found that SEEL30 induced cell death via apoptosis in dose dependent manners. SEEL30 had shown to have no reverse mutagenic potential on all bacterial strains tested.

**Conclusion**

SEEL30 and its biomarker EL30 were found to have anti-ovarian cancer potential awaiting to be validated via *in vivo* and pre-clinical studies.

**References**

[1] Nurhanan MY, et al. Cytotoxic effects of the root extracts of *Eurycoma longifolia* Jack. Phytotherapy Res. 2005, 19: 994-96.

[2] Skehan P, et al. New colorimetric cytotoxicity assay for anticancer-drug screening. J. Natl. Cancer Inst. 1990, 82(13): 1107-1112.

[3] Mahbub AA, et al. Differential effects of polyphenols on proliferation and apoptosis in human myeloid and lymphoid leukemia cell lines. AntiCancer Agents Med. Chem. 2013, 13:1601-1613.

### PP11 Protein profiling of A2780 ovarian cancer cell line treated with standardized extract of *Eurycoma longifolia*: towards the mode of action study

#### Nor Datiakma Mat Amin, Nurhanan Murni Yunos, Muhammad Hafiz Jauri and Noor Rasyila Mohamed Noor

##### Natural Products Division, Forest Research Institute of Malaysia (FRIM), Kepong, Malaysia

###### **Correspondence:** Nor Datiakma Mat Amin (nordatiakma@frim.gov.my)

**Background**

Ovarian cancer ranks fifth in cancer deaths among women and accounting for more deaths than any other cancer of the female reproductive system. At present, standard treatment for ovarian cancer involves the use of chemotherapy drugs paclitaxel and carboplatin after surgical to remove tumor to prolong patients’ life. However, the use of these drugs often leads to drug resistances that cause potential death of patients. Thus there is an urgent need for new drugs for ovarian cancer. Phytochemicals have huge chemical diversity and wide range of biological activities. Phytochemicals products have been used for thousand years as treatment for various diseases. Our previous studies discover that standardised extract of *E. longifolia* (SEEL30) have shown active in vitro anti-cancer effects against SKOV3 ovarian cancer cell line (UI2018703447). We aimed to profile the differentially expressed protein of A2780 ovarian cancer cell line after being treated with standardised extract of *E. longifolia* (SEEL30).

**Methodology**

Ovarian cancer cells (A2780) were treated separately with or without (control) standardised extract of *E. longifolia* (SEEL30) using IC50 value. Then, protein profiling was conducted using 2-Dimensional Electrophoresis (2-DE) (Nor Datiakma, 2010). The gels were stained with Coomassie Brilliant Blue-G200 (CBB-G200). Digital images of the analytical gels were acquired and analysed quantitatively for differentially expressed proteins using ImageMaster 2D Platinium 7.0 analysis software.

**Results and Discussion**

In total, at least 615 protein spots were successfully being separated in A2780 ovarian cancer cell line before they were subjected to SEEL30 treatment. Among them, 83 proteins were significantly altered in expression (up- or down-regulated) due to treatments with SEEL30 (selection criteria: Anova *p* value <0.05 and protein fold change >1.5). Of the 83 differentially expressed proteins, 63 proteins were up-regulated and 20 proteins were down-regulated.

**Conclusion**

Further work will be focusing on proteins identification and biochemical networks where these proteins are involved.

### PP12 Immune Regulation in the Development of Cervical Cancer

#### Shandra Devi Balasubramaniam, Venugopal Balakrishnan, Chern Ein Oon and Gurjeet Kaur

##### Institute for Research in Molecular Medicine (INFORMM), Universiti Sains Malaysia, 11800 Minden, Pulau Pinang, Malaysia

###### **Correspondence:** Gurjeet Kaur (gurjeet@usm.my)

**Background**

Cervical cancer is the fourth most common cancer in women. High risk human papillomavirus (HPV) type 16 and 18 are the main aetiological factors in cervical cancer. Persistent HPV infection causes a series of genetic alterations in the host cells, leading to transformation of normal cells into a precancerous stage termed cervical intraepithelial neoplasia (CIN), and further into cancer. The host’s immune system plays a critical role against viral infections and evading immune destruction, one of the hallmarks of cancer cells. This study aimed to identify the expression of HLA class II genes and other genes involved in the immune cell signalling pathways during cervical carcinogenesis.

**Methodology**

Total RNA was extracted from 12 formalin-fixed paraffin-embedded (FFPE) samples, consisting of three normal cervical tissues as control, three samples each of low-grade CIN (LGCIN), high-grade CIN (HGCIN) and squamous cell carcinoma (SCC). The extracted RNA was hybridized to Human Transcriptome Array (HTA) 2.0 Affymetrix. The expression of genes was determined using Affymetrix transcriptome analysis console software, using one-way ANOVA and *p* value set as < 0.05. The analysis compared the gene expressions between LGCIN and normal cervix, HGCIN and LGCIN, and SCC compared to HGCIN group.

**Results and Discussion**

The results showed that HLA-DRA and CD74 were significantly upregulated during transition from LGCIN to HGCIN. HLA-DRB4 was also found to be significantly upregulated in LGCIN. Invasion of HPV oncoprotein into the host cell initiates HLA class II. The main role of HLA class II in cervical precancerous lesions is to secrete proteins that facilitate immune recognition and control of HPV replication [1]. During the transition from HGCIN to SCC, IGHV3-15, IGHV3-21, and IGHV1-45 were found to be highly upregulated. In LGCIN, IGHD-21 was upregulated. The HGCIN stage is a crucial phase where genetic alterations significantly promote cell proliferation [2]. Immunoglobulins, which are normally produced by the B-cell lymphocytes, can also be derived from cancer epithelial cells. It has been demonstrated that these immunoglobulins promote cancer cell proliferation, invasion and metastasis, demonstrating its oncogenic potential [3]. The present study showed a high expression of immunoglobulins in SCC. Further studies are warranted to investigate the regulation of HLA genes and immunoglobulins in cervical cancer towards the future development of targeted immunotherapy.

**Conclusion**

HLA class II genes were activated in the early stages of transition from low grade to high grade CIN. Immunoglobulins were highly expressed in cancerous cells. These suggest that different immunological mechanisms are involved in precancerous and cervical cancer stages.

**Acknowledgement**

The work was funded by Research University Grant (No. 1001/CIPPM/812116) from Universiti Sains Malaysia.

**References**

[1] Lin P, Koutsky LA, Critchlow CW, Apple RJ, Hawes SE, Hughes JP, Touré P, Dembele A, Kiviat NB: HLA class II DR-DQ and increased risk of cervical cancer among Senegalese women. Cancer Epidemiol Biomarkers Prev 2001, 10:1037-1045.

[2] Niu G, Wang D, Pei Y, Sun L: Systematic identification of key genes and pathways in the development of invasive cervical cancer. Gene 2017, 618:28-41.

[3] Geng Z-H, Ye C-X, Huang Y, Jiang H-P, Ye Y-J, Wang S, Zhou Y, Shen Z-L, Qiu X-Y: Human colorectal cancer cells frequently express IgG and display unique Ig repertoire. World J Gastrointest Oncol 2019, 11:195

### PP15 Profiling and cytotoxicity study of *Xanthophyllomyces dendrorhous* extract from an astaxanthin overproducing mutant strain

#### Shin Yuan Khaw and Ai Lan Chew

##### Institute for Research in Molecular Medicine (INFORMM), Universiti Sains Malaysia, Penang, Malaysia

*BMC Proceedings* 2019, **Vol(Suppl no.):** P1

###### **Correspondence:** Ai Lan Chew (chew@usm.my)

**Background**

*Xanthophyllomyces dendrorhous* is a basidiomycetous yeast that has been biotechnologically exploited due to its ability to synthesize the carotenoid astaxanthin as its primary pigment and is a suitable producer for natural pigments with enhanced yield *via* modern biotechnological strategies. Astaxanthin has become increasingly important in food, cosmetic and pharmaceutical sectors because of its health-promoting benefits such as antioxidant, anti-diabetic and anticancer properties. Astaxanthin can be synthesized chemically but it is not environmental friendly (Charalampia *et al*., 2017). Meanwhile, the use of wild type *X. dendrorhous* in commercial-scale production has not been profitable as the pigment content in wild strains is low. So, an astaxanthin-overproducing *X. dendrorhous* strain, M34, was successfully isolated in our laboratory in view of the growing consumer demand for natural pigments obtained from renewable resources.

**Methodology**

Wild type *X. dendrorhous* (DSM 5626) and the mutant strain, M34, were grown in the yeast malt (YM) medium at 20°C with 200 rpm agitation for 96 h. The cell pellets were disrupted in the presence of glass beads and DMSO and extracted using petroleum ether. Both *X. dendrorhous* yeast extracts were spotted on a TLC sheet together with astaxanthin and beta carotene standards in a solvent system acetone: n-hexane with the ratio of 3:7 (v/v). HPLC analysis was performed on a reversed-phase column with Solvent A and Solvent B (9:1, v/v) as mobile phase with a flow rate of 1 mL/min. The elution spectra were detected using a diode array detector at 474 nm. Toxicity analysis was carried out on the immortalized but non-transformed human breast epithelial cells, MCF-10A. MTT assay was further conducted on the human breast cancer cell line (MDA-MB-231) in a dose-dependent manner to establish IC50 for both yeast extracts by software SigmaPlot 12.5.

**Results and Discussion**

Based on comparison to astaxanthin and β-carotene standards and results reported by Nagaraj *et al*. (2012), the TLC had shown well-separated bands corresponded to astaxanthin and β-carotene. HPLC analysis showed that the spectrum of M34 extract was in good agreement with the authentic standards in term of elution profile and retention time. The results identified that the major carotenoid produced by M34 was astaxanthin and in high purity as there was no other major carotenoid peak observed in the absorption spectrum. MTT assay showed that both the extracts of wild type and mutant strains were non-toxic towards the normalized breast cancer cells, MCF-10A, as the cell viability was over 80% at 72 hours with a maximum dosage of 30 μg/mL. Meanwhile, MTT assay on MDA-MB-231 cells (triple-negative human breast cancer cell line) gave IC50 of 10.06±0.63 ug/mL and 16.81±0.19 ug/mL for wild type and mutant extracts respectively. IC50<30 μg/mL indicated the high potential of a candidate compound for cancer therapy (Vijayarathna & Sasidharan, 2012).

**Conclusion**

The mutant yeast extract has been proven to be an alternative source of natural astaxanthin based on the profile study. It was also shown as a good candidate as an anticancer agent by inhibiting the MDA-MB-231 cancer cells at a low concentration.

**References**

[1] Charalampia *et al*,. (2017). Current trends and emerging technologies in biopigment production processes : Industrial food and health applications. International Journal of Horticulture, Agriculture and Food Science, 1(2), 33–46.

[2] Nagaraj *et al*,. (2012). Antiproliferative potential of astaxanthin-rich alga *Haematococcus pluvialis* Flotow on human hepatic cancer (HepG2) cell line. Biomedicine and Preventive Nutrition, 2(3), 149–153.

[3] Vijayarathna, S., & Sasidharan, S. (2012). Cytotoxicity of methanol extracts of Elaeis guineensis on MCF-7 and Vero cell lines. Asian Pacific Journal of Tropical Biomedicine, 2(10), 826–829.

### PP16 Modulation of macrophage activation marker induced by recombinant *Mycobacterium smegmatis* expressing Antigen-85B

#### Nur Ayuni Kadir^1^, Maria E Sarmiento^2^, Armando Acosta^2^, Mohd Nor Norazmi^2^

##### ^1^Faculty of Health Sciences, Universiti Sultan Zainal Abidin, Kampus Gong Badak, 21300 Kuala Terengganu, Malaysia; ^2^School of Health Sciences, Universiti Sains Malaysia, 16150 Kubang Kerian, Kelantan, Malaysia

*BMC Proceedings* 2019, **Vol(Suppl no.):** P1

**Background**

Tuberculosis (TB) remains a major worldwide health problem which causes more than 1.3 million deaths annually. The development of a new vaccine as a replacement of Bacille Calmette Guerin (BCG) or to improve its efficacy is one of the goals mooted by the World Health Organization (WHO) to control TB. *Mycobacterium smegmatis* (*M. smegmatis*) is nonpathogenic and commensal in humans, which shares many characteristics with *Mycobacterium tuberculosis* (*M. tuberculosis*). Mycobacterial vectors including *M. smegmatis* have been successfully used in the development of experimental vaccines against TB.

**Methodology**

Recombinant *M. smegmatis* expressing selected Ag85B epitopes (P1, P2, and P3) from *M. tuberculosis* was constructed (rMs064). As a control, *M. smegmatis* was also transformed with the empty plasmid (rMs012). The immunomodulatory effects of rMs064 were evaluated in J774A.1 murine macrophages cells. To test the effect of rMs064 infection on J774A.1 macrophage, the expression of macrophage activation markers; CD40, CD80 (B7.1) and CD86 (B7.2) and MHC-II were assessed by flow cytometry.

**Results and Discussion**

rMs064 was capable to induce expression of the macrophage activation markers; MHC class II and CD40 molecules. The expressions of co-stimulatory molecules followed by enhanced production of pro-inflammatory cytokines after macrophages were infected with rMs064. Our results showed enhanced expression of MHC-II and CD40 molecules after 24 hr post-infection. On the contrary, we demonstrated no significant increase of CD80 (B7.1) and CD86 (B7.2) expression post-infection with rMs064 or rMs012. Microbe-induced macrophage maturation is characterized by activation of antigen-presenting molecules such as MHCII and costimulatory activation markers such as CD40, CD80 (B7.1) and CD86 (B7.2) (Bhatt et al., 2009; Khan et al., 2012). The processing of exogenous antigens and its presentation results in the binding of peptide - MHC II complexes on the surfaces of APCs which is compulsory signal for T- cell receptor (TCR) activation (Holling et al., 2004). The second signal for further T cell activation is provided by co-stimulatory molecules B7 families and CD40.

**Conclusion**

The significant increase of MHC-II in rMs064 infected macrophages is an important indicator of efficient antigen processing and presentation. In conclusion, the immunomodulatory effects of rMs064 to initiate the innate immune response and activation of host macrophages make this vector as an efficient vehicle for further adaptive immune stimulation. The immunostimulating adjuvant modulated by *M. smegmatis* and the ability to express native *M. tuberculosis* antigens such as Ag85B, support further evaluation of this strain as an experimental TB vaccine candidate.

**References**

[1] Bhatt, K., Uzelac, A., Mathur, S., McBride, A., Potian, J., Salgame, P., 2009. B7 costimulation is critical for host control of chronic Mycobacterium tuberculosis infection. J. Immunol. 182, 3793–800.

[2] Holling, T.M., Schooten, E., Van Den Elsen, P.J., 2004. Function and regulation of MHC class II molecules in T-lymphocytes: Of mice and men. Hum. Immunol.65, 282-290.

[3] Khan, N., Gowthaman, U., Pahari, S., Agrewala, J.N., 2012. Manipulation of costimulatory molecules by intracellular pathogens: Veni, Vidi, Vici!! PLoS Pathog. 8, 1-7.

### PP17 Effects of Chrysin on mRNA Expression of PPARα and its Related Cellular Mechanisms in HCT116 Cells

#### Khor Chin Yin, Khoo Boon Yin

##### Institute for Research in Molecular Medicine (INFORMM), Universiti Sains Malaysia, Penang, Malaysia

###### **Correspondence:** Khor Chin Yin (chinyin81692@gmail.com)

**Background**

Peroxisome proliferator-activated receptor alpha (PPARα) is reported to regulate the transcription of cytochrome P450 genes in human liver and intestine, which accounts for 70-80% of the enzymes involved in drug metabolism [1,2]. Chrysin is a natural phytochemical flavonoid that may possess a mild effect to be used as an anticancer agent [3]. This study aimed to investigate the drug metabolising effect of Chrysin by investigating the mRNA expression level of PPARα and its related cellular mechanisms in HCT116.

**Methodology**

The mRNA expressions of PPARα in HCT116 treated with Chrysin or combination of Chrysin and MK886 were first determined using PCR and quantitatively analysed by Bioanalyzer. Each quantified level of mRNA expression was then normalised to GAPDH. Flow cytometry was used to detect the cell cycle phases of HCT116 treated with Chrysin or combination of Chrysin and MK886. The migration of HCT116 post treatment with Chrysin or combination of Chrysin and MK886 were also investigated by Wound Healing assay. Finally, the mRNA expression of metabolism-related CYP genes in Chrysin or combination of Chrysin and MK886 treated HCT116 were determined by PCR.

**Results and Discussion**

The mRNA expression of PPARα was significantly induced in HCT116 following treatment with Chrysin for 36 hours, indicating that Chrysin-treated HCT116 was able to regulate the transcription of cytochrome P450. The mRNA expression of PPARα was inhibited when the cells were treated with a combination of Chrysin and MK886 (PPARα inhibitor) proven that the incorporation of MK886 lowers the expression level of PPARα, thus enabling us to study the function of PPARα. The cell population of the G0/G1 phase was detected significantly increased in Chrysin-treated cells, which was accompanied by a decrease in the percentage of S phase cell population at 12 hours of treatment. However, treatments of HCT116 with Chrysin or combination of Chrysin and MK886 did not show the opposite situation in the G0/G1 and S-phase cell populations, indicating that the expression of PPARα may not be associated with the cell cycle in the treated cells. The migration rate in Chrysin treated HCT116 were reduced significantly at 24 and 36 hours of treatments. However, the activity was revived when the expression of PPARα was inhibited, indicating that the migration of Chrysin-treated cells is likely correlated with the expressions of PPARα, CYP2S1 and CYP1B1 in the cells, whereby the expression of CYP genes could be modified by the PPARα inhibitor.

**Conclusion**

Treatment of HCT116 with Chrysin induced the expression of PPARα, which could be attenuated by administrating a combination of Chrysin with MK886 to the cells. The expression of PPARα in HCT116 is likely correlated with cell migration and the expressions of CYP2S1 and CYP1B1.

**References**

[1] Thomas, et al. (2013). Direct transcriptional regulation of human hepatic cytochrome P450 3A4 (CYP3A4) by peroxisome proliferator-activated receptor alpha (PPARα). Mol. Pharmacol. 83, 709–718.

[2] Thomas, et al. (2015). Peroxisome proliferator-activated receptor alpha, PPARα, directly regulates transcription of cytochrome P450 CYP2C8. Front Pharmacol. 6.

[3] Khoo, et al. (2010). Apoptotic Effects of Chrysin in Human Cancer Cell Lines. Int. J. Mol. Sci. 11, 2188–2199.

### PP18 The immuno-binding of graphene quantum dots-conjugated antibody to *X.oryzae pv. oryzae* cells

#### Norhafniza Awaludin^1,2^, Jaafar Abdullah^2,3^, Faridah Salam^1^, Nor Azah Yusof^2,3^, Helmi Wasoh^3^, Kogeetahavani Ramachandran^4^, Rafidah Abd Rahman^1^ and Nurul Hidayah Hussin^1^

##### ^1^Biotechnology and Nanotechnology Research Centre, Malaysian Agricultural Research and Development Institute, Serdang, Selangor, Malaysia; ^2^Institute of Advanced Technology, University of Putra Malaysia (UPM), Serdang, Selangor, Malaysia; ^3^Faculty of Science, University of Putra Malaysia (UPM), Serdang, Selangor, Malaysia; ^4^Rice Research Centre, MARDI Seberang Perai, Pulau Pinang, Malaysia.

###### **Correspondence:** Norhafniza Awaludin (hafniza@mardi.gov.my)

**Background**

Bacterial leaf blight disease (BLB) is one of the major diseases in rice crop caused by a gram-negative bacterium; *Xanthomonas oryzae pv. oryzae (Xoo)*[1]. The detection of BLB typically proceeds by plate culturing method that has drawback issues on time-consuming and late-stage detection. The development of biosensor technologies nowadays is an alternate approach for the rapid detection of plant disease pathogens [2]. Optical fluorescence biosensor using nanomaterials have drawn much attention as becoming a promising field for various analyte detection. The use of antibody as biorecognition molecule is enabled to recognize and interact with target cells via high specificity binding. The conjugation of antibody with graphene quantum dots (GQDs) nanoparticle is an important probe of fluorescence-based optical immunosensor for *Xoo* detection. In this paper, we present a study of immuno-recognition binding of GQDs conjugated antibody towards *Xoo* cells via high-resolution transmission electron microscopy analysis.

**Methodology**

The polyclonal antibody against whole cells of Xoo was produced by subcutaneously approach in New Zealand white rabbits. The produced antibody (AntiXoo) was conjugated with GQDs by carbodiimide conjugation method. The 50 μL of bioprobe (AntiXoo-GQDs) was incubated with 20 μL of Xoo cells (10^3^ CFU mL^-1^) in 200 μL of total volume with 0.01M PBS buffer (pH7.4) for 2 hours at 4°C. Subsequently, 50 μL of the immunoreaction sample was proceeded for coating, staining and drying to be visualized by high-resolution transmission electron microscopy (HRTEM) at accelerating voltage 200kV.

**Results and Discussion**

The AntiXoo antibody was successfully produced and achieved high titer at 1:100000 of dilution ratio. The antibody was highly specific to *Xoo* cells and the low cross-reaction was observed with the other plant pathogens via indirect enzyme-linked immunosorbent (ELISA) assay. From the HRTEM microscopic analysis, the AntiXoo-GQDs probe was highly specific bound to the *Xoo* cells at two antigenic sites; cell surface (outer membrane) and flagellum structure. It describes the interaction of AntiXoo-GQDs probe to antigenic sites that high probability contained lipopolysaccharide (LPS) as the main antigenic properties of the outer membrane that essential for cellular stability and pathogenicity to invade plant hosts [3].

**Conclusion**

The high immunorecognition binding of AntiXoo-GQDs to the specific antigenic sites has enhanced the superiority of bioprobe in optical-based immunosensor for the rapid detection of *X.oryzae pv. oryzae*.

**References**

[1] Jonit, N.Q., Low, Y.C. & Tan, G.H. 2016. *Xanthomonas oryzae pv. oryzae*, biochemical tests, rice (Oryza sativa), bacterial leaf blight (BLB) disease, Sekinchan. J. Appl. Environ. Microb. 4(3): 63-69

[2] Khater, M., Escosura-Muniz, A.d.l. & Merkoci, A. 2017. Biosensor for plant pathogen detection. BioSens Bioelectron. 93: 72-86

[3] Steffens, T., Duda, K., Lindner, B., Vorholter, F.J., Bednarz, H., Niehaus, K. & Holst, O. 2017. The lipopolysaccharides of the crop pathogen *Xanthomonas lipopolysaccharide pv. translucen*s: chemical characterization and determination of signaling events in plant cells. Glycobiology. 27(3): 264-274

### PP21 Body Mass Index (BMI) and CA125 as the Predictive Biomarkers to the Protein Expression of PTEN and PI3K in Endometrioid and Clear Cell Carcinoma of the Ovary in Women with Underlying Endometriosis

#### Abdul Muzhill Hannaan Abdul Hafizz^1,2^, Noorazizah Arsad^1^, Nur Maya Sabrina Tizen Laim^2^, Reena Rahayu Md Zin^2^, Nor Haslinda Abd Aziz^1^ and Mohamad Nasir Shafiee^1^

##### ^1^Department of Obstetrics and Gynaecology, Faculty of Medicine University Kebangsaan Malaysia Medical Centre; ^2^Department of Pathology, Faculty of Medicine University Kebangsaan Malaysia Medical Centre.

###### **Correspondence:** Mohamad Nasir Shafiee (nasirshafiee@hotmail.com)

**Background**

Ovarian cancer is the most common gynecological cancer in women worldwide; it also known to be associated with BMI and CA125, especially those with underlying endometriosis. The PTEN and PI3K in endometriosis also widely studied, however their roles in the endometrioid adenocarcinoma and clear cell carcinoma of ovary are still unclear. Our study aims to evaluate the BMI and CA125 level with the expression level of PTEN and PI3K in endometrioid adenocarcinoma and clear cell carcinoma of the ovary in women with underlying endometriosis.

**Methodology**

Tissue microarray (TMA) were constructed from 19 paraffin blocks of ovarian endometrioid and clear cell carcinomas, collected between 2007 until 2015 from the Diagnostic Laboratory, University Kebangsaan Malaysia Medical Centre (UKMMC). Immunohistochemical staining (IHC) for PTEN and PI3K were done on tissue sections that reported with ovarian cancer with endometriosis (n=10) vs. ovarian cancer without endometriosis (n=9) following analysis of each patient’s medical record. BMI and CA125 records were taken for analysis.

**Results and Discussion**

In ovarian cancer with underlying endometriosis, we found greater loss of PTEN protein expression and higher PI3K expression compared to those without endometriosis but were not statistically significant (p>0.05); 100% vs. 88.9% and 80% vs. 77.8% respectively. In addition, BMI was not significantly (p>0.05) cause loss of PTEN and phosphorylation of PI3K in both groups. CA125 was higher in those without endometriosis; it also increased in absent of PTEN and higher of PI3K but not significantly different (p>0.05). In this study, we found endometriosis were not responsible for the elevated CA125 level.

**Conclusion**

The present study demonstrates the phosphorylation of PI3K accompanied by the loss of PTEN in clinical specimens of ovarian endometrioid adenocarcinoma and clear cell carcinoma with endometriosis. CA125 might be used to predict PTEN and PI3K expression.

### PP27 Expression of Soluble Recombinant *Brucella abortus* Outer Membrane Protein

#### Basyirah Ghazali^1^, Nur Eqa Mardihah Che Anuar^2^, Nik Abdul Aziz Nik Kamarudin^1^, Che Muhammad Khairul Hisyam Ismail^1^, Chiuan Yee Leow ^1^ and Khairul Mohd Fadzli, Mustaffa^1^

##### ^1^ Institute for Research in Molecular Medicine (INFORMM), Health Campus, Universiti Sains Malaysia, Malaysia; ^2^ Faculty of Science and Marine Environment, Universiti Malaysia Terengganu, Malaysia.

###### **Correspondence:** Khairul Mohd Fadzli, Mustaffa (khairulmf@usm.my)

**Background**

Brucellosis is particularly dangerous and highly contagious zoonotic disease which is transmitted from ruminants to human. Four of them are known to infect human (*B. melitensis, B. abortus, B. canis, B. suis*) and the most virulent strain that pathogenic to human are *B.melitensis* and *B.abortus* [1,2]*.* Currently, the diagnosis of brucellosis is depending on isolation and serological techniques. Therefore, outer membrane protein from *B. abortus* strain was selected as a target for diagnostic and vaccine development. This strain was known can infected broad host target compared to another three Brucella strains. Thus, this study was aimed to produce recombinant outer membrane protein from *B. abortus* as a target for vaccine and diagnostic development.

**Methodology**

The plasmid pET-28-a(+) containing *B. abortus* outer membrane protein (namely as 3BP) was transformed into *E. coli* BL21-DE3. The positive transformed was inoculated into LB broth for overnight culture. The overnight culture was diluted 1/100 into expression media (10 mL of LB media with 1% glucose or 10 mL of 2xYT media with 0.2% glucose) and induced with 1 mM IPTG when OD600 reaches 0.5-0.6. After induction, the cultures were incubated at 37°C at different speed (200 rpm and 120 rpm) and incubation time. The protein pellet was lysed with lysis buffer. The collected supernatants containing total protein extract were measured using NanoDrop 100 Spectrophotometer. The recombinant protein expression was analyzed using SDS-PAGE and western blot.

**Results and Discussion**

The total protein expressed in LB and 2xYT media was measured at 4, 5 and 16 hours. The yield of total protein expressed in LB media was measured at 143.8 mg (4 hrs, 200 rpm), 130.4 mg (5 hrs, 200 rpm) and 156.6 mg (16 hrs, 120 rpm). Meanwhile, in 2xYT media, the yield of total protein expressed in 2xYT media was measured at 280.5 mg (4 hrs, 200 rpm), 305.3 mg (5 hrs, 200 rpm) and 287.3 mg (16 hrs, 120 rpm). The SDS-PAGE and western blot were confirmed the 3BP protein at ~15 kDa present in both pellet and supernatant.

**Conclusion**

This study was successfully expressed the recombinant of outer membrane protein (namely as 3BP) from *B. abortus* which could potentially be used as a target for diagnostic application. However, further optimization is needed to increase protein solubility before the protein can be purified.

**References**

[1] He Y: Analyses of Brucella Pathogenesis, Host Immunity, and Vaccine Targets using Systems Biology and Bioinformatics. Front Cell Infect Microbiol 2012, 2.

[2] Wang Q, Zhao S, Wureli H, Xie S, Chen C, Wei Q, Cui B, Tu C, Wang Y: Brucella melitensis and B. abortus in eggs, larvae and engorged females of Dermacentor marginatus. Ticks and tick-borne diseases 2018, 9(4):1045-1048.

### PP28 Optimization of Single-stranded DNA Production using Asymmetric PCR Coupled with Streptavidin-conjugated Resin

#### Nur Fatihah Mohd Zaidi^1^, Asmadamia Abdul Aziz ^2^, Nik Abdul Aziz Nik Kamarudin^1^, and Khairul Mohd Fadzli, Mustaffa^1^

##### ^1^ Institute for Research in Molecular Medicine (INFORMM), Health Campus, Universiti Sains Malaysia, Malaysia; ^2^ Faculty of Science and Marine Environment, Universiti Malaysia Terengganu, Malaysia.

###### **Correspondence:** Khairul Mohd Fadzli, Mustaffa (khairulmf@usm.my)

**Background**

Aptamer is a single-stranded nucleic acid (DNA or RNA) that is valuable in disease diagnostics and therapeutics due to its ability to bind to a various molecule with high specificity and affinity [1]. In term of DNA aptamer, the most critical step is during the production of single-stranded DNA (ssDNA) that has high yield and purity. There is a lot of methods that have been reported previously for ssDNA production including lambda exonuclease, NaOH denaturation, magnetic beads, and polyacrylamide gel passive elution [2]. However, some of the methods either give low yields of ssDNA or require a lot of resources [3]. Thus, this study was aimed to develop a method of ssDNA production using asymmetric PCR coupled with a streptavidin-conjugated resin that acts as dsDNA capturing agent.

**Methodology**

Asymmetric PCR was performed to amplify the 83-mers single-stranded DNA library by utilizing various amounts of unmodified forward and biotinylated reverse primer ratio using the previously optimized PCR condition. Further optimization of primer ratio was performed by fixing the amount of biotinylated reverse primer, but with the variation of unmodified forward primer ratio by 1-fold dilution. The optimization of the PCR cycle was performed at 15, 20, 25, and 30 cycles in order to determine the effect of number PCR cycle on variability of PCR products. The bulk library amplification was conducted using optimized asymmetric PCR condition prior to ssDNA purification. The streptavidin-conjugated resin was added into the PCR product to capture the biotinylated dsDNA. The supernatant containing ssDNA was collected after pellet down the resin containing biotinylated dsDNA and ethanol precipitated. The obtained ssDNA was measured by using NanoDrop 1000 Spectrophotometer and confirm the purified by agarose gel.

**Results and Discussion**

Amplification of single-stranded DNA (ssDNA) was successfully obtained from asymmetric PCR with different amounts of forward and reverse primer. The utilization of different primers amounts preferential increased the amplification of targeted ssDNA, however, also increased the production of primer excess. Fixation of reverse primer, with various forward primer ratio, was showed to decrease the production of primer excess compared to the previous amplification with different amounts of both primers, but with the increasing of ssDNA production. The optimization of PCR cycle with primer ratio of 1.5:0.2 (forward:reverse) showed the appearance of non-specific binding after 15 cycles.

**Conclusion**

This study was successfully optimized the asymmetric PCR for ssDNA production and minimize the dsDNA present in PCR product. Further purification using streptavidin-conjugated resin was successfully removed the dsDNA from ssDNA product. However, further optimization is needed to eliminate the excess primer contamination without affecting the yield of ssDNA.

**References**

[1] Nik Kamarudin N, Mohammed N, Mustaffa K: Aptamer Technology: Adjunct Therapy for Malaria. Biomedicines 2017, 5(1):1.

[2] Marimuthu C, Tang T-H, Tominaga J, Tan S-C, Gopinath SCB: Single-stranded DNA (ssDNA) production in DNA aptamer generation. The Analyst 2012, 137(6):1307-1315.

[3] Svobodová M, Pinto A, Nadal P, O’ Sullivan CK: Comparison of different methods for generation of single-stranded DNA for SELEX processes. Analytical and bioanalytical chemistry 2012, 404(3):835-842.

